# Characteristic gene expression profiles in the progression from liver cirrhosis to carcinoma induced by diethylnitrosamine in a rat model

**DOI:** 10.1186/1756-9966-28-107

**Published:** 2009-07-29

**Authors:** Yue-Fang Liu, Bin-Shan Zha, Hui-Lin Zhang, Xiao-Jing Zhu, Yu-Hua Li, Jin Zhu, Xiao-Hong Guan, Zhen-Qing Feng, Jian-Ping Zhang

**Affiliations:** 1 Department of Pathology, Nanjing Medical University, 140 Han Zhong Road, Nanjing 210029, PR China; 2 Key Laboratory of Antibody Technique of Ministry of Health, Nanjing Medical University, 140 Han Zhong Road, Nanjing 210029, PR China; 3 Department of General Surgery, Second Affiliated Hospital of Nanjing Medical University, 121 Jiang Jia-Yuan Road, Nanjing 210011, PR China

## Abstract

**Background:**

Liver cancr is a heterogeneous disease in terms of etiology, biologic and clinical behavior. Very little is known about how many genes concur at the molecular level of tumor development, progression and aggressiveness. To explore the key genes involved in the development of liver cancer, we established a rat model induced by diethylnitrosamine to investigate the gene expression profiles of liver tissues during the transition to cirrhosis and carcinoma.

**Methods:**

A rat model of liver cancer induced by diethylnitrosamine was established. The cirrhotic tissue, the dysplasia nodules, the early cancerous nodules and the cancerous nodules from the rats with lung metastasis were chosen to compare with liver tissue of normal rats to investigate the differential expression genes between them. Affymetrix GeneChip Rat 230 2.0 arrays were used throughout. The real-time quantity PCR was used to verify the expression of some differential expression genes in tissues.

**Results:**

The pathological changes that occurred in the livers of diethylnitrosamine-treated rats included non-specific injury, fibrosis and cirrhosis, dysplastic nodules, early cancerous nodules and metastasis. There are 349 upregulated and 345 downregulated genes sharing among the above chosen tissues when compared with liver tissue of normal rats. The deregulated genes play various roles in diverse processes such as metabolism, transport, cell proliferation, apoptosis, cell adhesion, angiogenesis and so on. Among which, 41 upregulated and 27 downregulated genes are associated with inflammatory response, immune response and oxidative stress. Twenty-four genes associated with glutathione metabolism majorly participating oxidative stress were deregulated in the development of liver cancer. There were 19 members belong to CYP450 family downregulated, except CYP2C40 upregulated.

**Conclusion:**

In this study, we provide the global gene expression profiles during the development and progression of liver cancer in rats. The data obtained from the gene expression profiles will allow us to acquire insights into the molecular mechanisms of hepatocarcinogenesis and identify specific genes (or gene products) that can be used for early molecular diagnosis, risk analysis, prognosis prediction, and development of new therapies.

## Background

HCC is a heterogeneous disease in terms of etiology, biologic and clinical behavior. Meanwhile, hepatocarcinogenesis is a long-term, multistep process associated with changes in gene expression profiles. In the last several years, there have been important gains in our understanding of the pathogenesis of HCC and our appreciation of the critical oncogenic and tumor suppressor pathways involved in hepatocarcinogenesis [1-5]. Despite this, current knowledge about the molecular pathogenesis of HCC is a result of investigations of fully developed HCC. Very little is known about how many genes concur at the molecular level of tumor development, progression and aggressiveness.

Molecular profiling has been successfully used to identify candidate genes for HCC in human and animal model systems[3]. Although many approaches (including genome-scale studies) provide insights into some of the stages in human tumorigenesis, a sequential analysis of the development of tumors in humans is very difficult. Most of them have not given us the gene expression profiles that could point to those genes that play key roles during the whole carcinogenetic process from initiation to metastasis. Animal models of carcinogenesis have permitted the examination of the stages of neoplastic development in considerable detail.

In this study, we established the rat model of liver cancer induced by DEN to explore the processes of initiation and progression of HCC[6]. HCC develops commonly, but not exclusively, in a setting of chronic liver cell injury, which leads to inflammation, hepatocyte regeneration, liver matrix remodeling, fibrosis, and ultimately, cirrhosis[7,8]. The histological changes in DEN-induced liver cancer in rats are similar to those seen in human HCC. We think the similar phenotype might be based on similar gene expression profiles. Affymetrix GeneChip Rat 230 2.0 arrays were used to analyze gene expression profiles of liver tissues from control and DEN-treated rats during the process from cirrhosis to metastasis. This allowed us to obtain an almost complete picture of the early genetic alterations that are directly or indirectly involved in the development of HCC. We supposed that the genes expression profiles deregulated during the process from liver cirrhosis to carcinoma and metastasis play key roles in the hepatocarcinogenesis. The data obtained from the gene expression profiles will allow us to acquire insights into the molecular mechanisms of hepatocarcinogenesis and identify specific genes (or gene products) that can be used for early molecular diagnosis, risk analysis, prognosis prediction, and development of new therapies.

## Materials and methods

### Animals and treatments

Male Wistar rats weighing 120–150 g at the beginning of the experiments were obtained from SLAC Laboratory Animal Co. Ltd. (Shanghai). The animals were acclimatized to standard laboratory conditions (temperature 22–25°C, relative humidity 50–60%, and 12 hour photoperiods (lights on 07:00–19:00 h)) and were housed in stainless steel wire-mesh cages (five rats per cage). During the entire period of study, the rats were supplied with a semi-purified basal diet (Shanghai) and water ad lib. All experiments followed the Guide for the Care and Use of Laboratory Animals.

Briefly, ninety Wistar rats were randomly divided into two groups: the control and DEN group (DEN, Sigma Chemical Co. St Louis, MO; CAS 56-23-5; purity > 98%). After one week on a basal diet, rats belonging to the DEN group underwent intragastric administration of 1% aqueous solution of DEN (70 mg/kg) once a week, consecutively for 14 weeks. Animals that belonged to the control group received distilled water. There were ten rats in the control group. Daily food and water intakes were noted and the body weights of the animals from each group were recorded every second day. The rats were sacrificed with 25% (g/ml) urethane anesthesia (6 ml/kg) by intraperitoneal injection and sacrificed at different time points. At the end of the 2^nd^, 4^th ^and 6^th ^week after DEN-treatment, 3, 3, and 4 rats were sacrificed respectively at these time periods. From the end of the 8^th ^week until the end of the 20^th ^week after DEN-treatment, ten rats were sacrificed respectively every two weeks. Meanwhile one age-matched control rat was sacrificed. All the age-matched rats from the control group and rats of treatment groups were sacrificed on the same day.

### Sample collection and histopathological analyses

Animals were chosen sequentially for necropsy. The entire liver was observed grossly and weighted. The selected liver tissues were observed for gross changes, divided into pieces of about 0.1 g, snap-frozen directly in liquid nitrogen and stored at -80°C prior to RNA isolation for microarray analysis. The remaining livers were preserved in 10% phosphate-buffered formalin. The liver tissue fixed in neutral formalin was embedded in paraffin, sectioned, and stained with hematoxylin and eosin (H&E). Histopathologic examinations of the liver sections were conducted by a pathologist and peer-reviewed.

### RNA extraction

Frozen liver sections were ground in a Mixer Mill mm 200 (Retsch GmbH and Co. KG, Haan, Germany) using pre-cooled stainless steel balls. Total RNAs were isolated from livers with Trizol Reagent (Invitrogen, CA) using manufacturer recommended procedures. The ratio of the optical densities from RNA samples measured at 260 and 280 nm was used to evaluate nucleic acid purity, and total RNA concentrations were determined by the absorbance at 260 nm. The quality of total RNA was estimated based on the integrity of 28S and 18S rRNA. RNA was separated using 1% agarose gel electrophoresis. Good RNA quality was indicated by 28S rRNA banding having twice the intensity of the 18S rRNA, without significant smearing of the rRNA bands. Samples of total RNA from livers of rats from the same time points were pooled for subsequent use in the GeneChip analysis. Prior to GeneChip analysis, the pooled total RNA samples were purified using the RNeasy Total RNA Mini Kit (Qiagen, Valencia, CA) according to manufacturer's instructions.

### Oligo microarray hybridization

Biotin-labeled cRNA samples were used for hybridization of Affymetrix GeneChip Rat 230 2.0 arrays. The arrays were prepared according to the protocol supplied with the GeneChip Sample Cleanup module (P/N 900371, Affymetrix Inc., Santa Clara, CA). Briefly, 5 μg total RNA was used for cDNA synthesis with the SuperScript Choice System (Invitrogen Life Technologies, Carlsbad, CA) employing a T7-(d7)_24 _primer. After spin column purification, biotin-labeled cRNA was synthesized from the cDNA using the ENZO RNA Transcript Labeling Kit (Affymetrix Inc.). Spin column-purified cRNA was quality controlled using an Agilent 2100 Bioanalyzer and spectrophotometrically quantified. The cRNA (15 μg) was then fragmented in buffer supplied with the Cleanup Module and hybridized for 16 h at 45°C (Affymetrix Genechip Hybridization Oven 640). The microarrays were washed and stained with streptavidin-phycoerythrin (SAPE, Molecular Probes) on the Affymetrix Fluidics Station 450, including an amplification step according to the manufacturer's instructions. Fluorescent images were read using the Gene Array Scanner 3000. The raw data image files (DAT) were converted into RPT files using Affymetrix Microarray Suite (MAS) 5.0. In RPT files, the scan data from the 36 pixels per oligo set were averaged. Pooled RNAs of cirrhotic tissue, the dysplasia nodules, the early cancerous nodules and the cancerous nodules from the rats with lung metastases at the 12^th^, 14^th^, 16^th^, and 20^th ^week after DEN-treatment and from normal control rat were processed, hybridized and analyzed individually.

The chips were scanned in an Agilent ChipScanner to detect hybridization signals. Average target intensity was set at 500 arbitrary units. Each array was assessed for quality and stability by examining replicated copies of the same gene at different locations on the array. To ensure the quality of the cRNA samples and of the Affymetrix GeneChips, quality control experiments were performed using test chips, and the same cRNA sample used in both the test chip and GeneChip.

### Microarray quality control

With GeneChip Operating Software (GCOS) v1.2, dark and white spots, gradients and distortions were detected and corrected using the RPT file data. The GeneChip Rat Genome 230 2.0 Array provides the entire transcribed rat genome on a single array and enables scientists to obtain the most comprehensive view of the transcribed rat genome in order to make accurate biological conclusions. The Affymetrix Rat Genome 230 2.0 microarrays contain 31,000 probe sets corresponding to about 24,000 annotated rat genes and 6693 expressed sequence tags (ESTs). Each probe set is represented by 11–20 pairs of 25 mer oligonucleotides. Each probe pair consists of a perfect match oligo (PM) complementary to the cRNA target sequence and a mismatched oligo (MM). Using the MAS 5.0 statistical algorithms implemented in the Quality Controller software, the intensities of all 11–20 probe pairs were condensed to one intensity value per probe set associated with a statistical detection *p *value calculated from the intensity differences of the PM and corresponding MM oligos. This *p *value indicates how reliably a transcript is detected. Transcripts with p < 0.04 were designated present, whereas those with a p > 0.06 were designated absent. Transcripts with 0.04 < p < 0.06 were designated marginal, whose reliability were doubted and need to be verified by methods with higher sensitivity. After condensing (which also included overall microarray background correction) the microarray was scaled to an average signal intensity of 100, after excluding the highest and lowest 2% of the data. GeneChip Rat Genome 230 2.0 microarrays include a set of rat maintenance genes to facilitate the normalization and scaling of array experiments. These probe sets serve as a tool to normalize or scale data prior to performing data comparison. These normalization genes show consistent levels of expression over defined sample sets.

### Microarray data analysis

The microarray data were analyzed using the microarray suite 5.0 software. First, using the present genes, those significantly deregulated between the DEN-treated and control groups were selected using a two-sample t-test with a p cutoff value of 0.001 in combination with n-fold regulation/ratio of means. This requires that the mean intensity of a gene has to be at least n times different between the treated and control sample group to be included. The cutoff was set at 2 times.

Secondly, genes designated present in treated samples but absent in controls, or vice versa, were determined, as these could be genes induced from or repressed to background expression levels, respectively, after treatment. From these genes, those discriminating between treated and control samples were again selected with a two-sample t-test (p < 0.001), combined with the requirement of an at least two-fold difference of the mean intensities for a given gene.

### Scatter plot, gene tree

Scatter plots were used to visually examine the expressional level of genes between the control and DEN-exposed groups. Hierarchical dendrograms were drawn with the Cluster (2.0). It was created by clustering the genes according to their expression in response to the carcinogenic agent. Genes sharing similar expression profiles tended to be clustered together, and the location of a branch containing the genes can be considered a measure of how similar the gene expression was. Genes were selected for the construction of gene tree if the expression of the gene was two-fold greater or less in the treatments, relative to that in the corresponding control. The horizontal axis shows the clustering of the genes according to their expression across treatments; while the vertical axis showed the clustering according to their expression profile in the treatment.

### Statistical analysis

The genechip probe array system only allows comparison of one treatment hybridizing with the probe set. In a comparison analysis, two samples were hybridized to two genechip probe arrays of the same type, they were compared against each other in order to detect and quantify changes in gene expression. One genechip was for baseline (control) and the other was for the experiment (treatment). Two sets of algorithms were generated and they were used to generate change significance and change quantity metrics for every probe set using Microarray Suite (MAS) version 5.0 (Affymetrix, CA). The change algorithm generated a Change p value and an associated fold-change value. The second algorithm gave a quantitative estimate of the change in gene expression in the form of Signal Log Ratio. In the present study, the level of gene expression can be regarded as increased if its Change p-value was less than 0.002 and the gene expression would be considered to be decreased if its Change p-value was greater than 0.997. This method has been used by other investigators. Fold change could be calculated with the following formula: fold change = 2^(signal log ratio)^.

### Validation of differential expression of genes by real-time RT-PCR

The differential expression of selected genes was further validated by real-time PCR with SYBR green-based detection (ABI) using gene-specific primer pairs that were run on an ABI 7000 fluorescent sequence detection system (Perkin-Elmer, Foster City, CA). It is known that only the cDNA of each target gene can be the template, owing to the fact all the primer sequences span one or more introns by design (Gene Runner). The rat housekeeping gene β-actin was used as the control. Quantitative values were obtained from the cycle number (Ct value) at which the increase in fluorescent signal (associated with exponential growth of PCR products) starts to be picked up by the laser detector of the detection system. Results, expressed as N-fold differences in target gene expression between the liver tissues of DEN-treated and normal rats and termed 'Ntarget' were determined using the formula: Ntarget = 2^ΔCtsample ^(while ΔCt_sample _= ΔCt_DEN _- ΔCt_Normal_), where the ΔCt_DEN _and ΔCt_normal _values of the sample were determined by subtracting the Ct value of the target gene from the average Ct value of the β-actin gene.

## Results

### Histopathology

**Figure 1:**
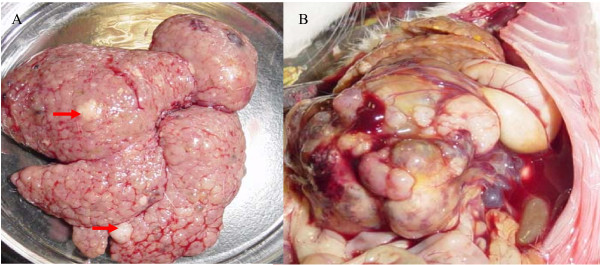
**The gross appearance of the livers from DEN-treated rats**. (A-B) The liver from the rat by DEN-treated at the 16^th ^week (red arrows stick to early cancerous nodules(A); The metastasis mass in the abdominal cavity from the rat by DEN-treated at the 20^th ^week (B).

**Figure 2:**
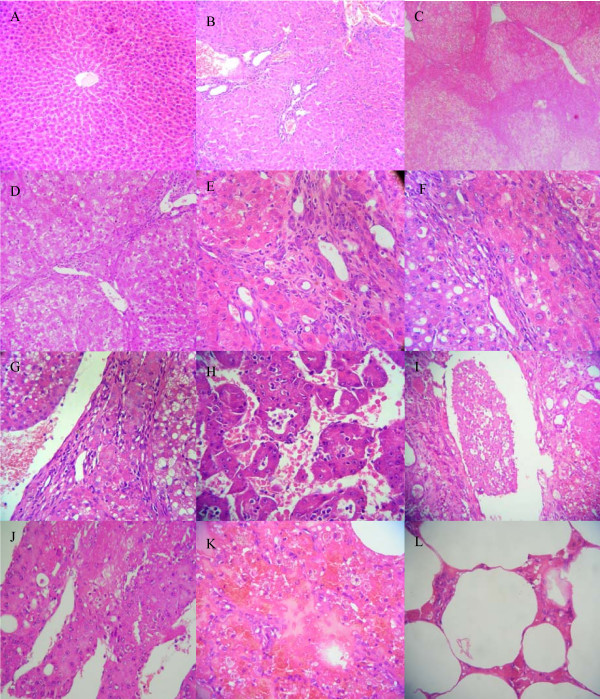
**The histological changes of livers from control and DEN-treated rats**. (A) the normal liver tissue from rat of control group; (B-L) tissures from rats by DEN-treated: (B) non-special injury of liver at the 6^th ^week; (C) liver fibrosis at the 8^th ^week; (D) liver cirrhosis at the 10^th ^week; (E) liver cirrhosis rat at the 12^th ^week; (F) dysplasia nodules at the 14^th ^week; (G) liver carcinoma at the 16^th ^week; (H) liver carcinoma at the 20^th ^week; (I) tumor embolism in blood vessel at the 20^th ^week; (J) the metastasis mass in the abdormainal cavity at the 20^th ^week; (K) lung metastasis at the 20^th ^week; (L) lung tissure of normal rat.

The histological changes of livers of the DEN-treated rats can be divided into three stages. Initially, from the 2^nd ^to 8^th ^week, non-specific injury occurred such as cellular swelling, fatty changes, necrosis, inflammatory infiltration and hepatocyte regeneration. On the 10^th ^to the 14^th ^week, significant liver fibrosis occurred. At the 10^th ^week, the livers showed an quantitative increase in connective tissue, and encapsulation of regenerative nodules, while at the end of the 12^th ^week, nodular cirrhosis could be seen macroscopically. At the 14^th ^week, gray-white nodules, 3 mm to 5 mm in diameter, could be distinguished from the surrounding reddish brown cirrhosis nodules in the livers of 2/10 rats. These were histologically diagnosed as dysplastic nodules. From the 16^th ^to the 20^th ^week the number of nodules increased significantly. At the 16^th ^week, nodules, 5 mm to 1.5 cm in diameter, could be distinguished in the livers of 8/10 rats, while at the 18^th ^and the 20^th ^week, gray-white nodules were present in the livers of all 20 rats. In addition, by the 20^th ^week, abdominal cavity and lung metastases were observed in 2/10 rats. (Figure 1, 2)

### Isolation of RNA, microarray hybridization, scanning and signal detection

**Figure 3:**
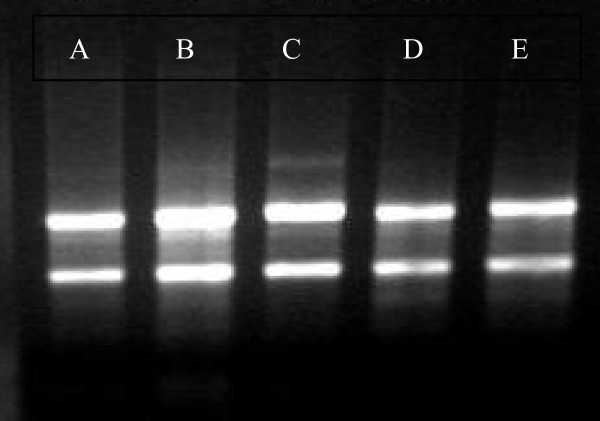
**Total RNA isolated from the liver tissues of the rats was identified by agar electrophoresis**. (A) from normal rats; (B-E) from DEN-treated rats: cirrhosis tissue at 12^th ^week (B), dysplastic nodules at the 14^th ^week (C), early cancerous nodules at the 16^th ^week (D), cancerous nodules with lung metastasis at the 20^th ^week (E).

**Figure 4:**
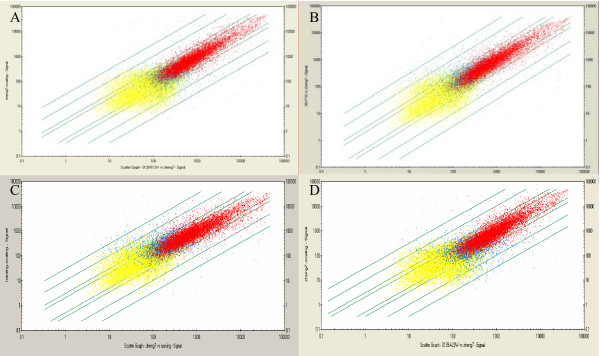
**Scatter plot of gene expression comparisons between the normal rats and DEN-exposured rats**. Each point represents a single gene or EST. x-axis: control (from liver tissue of normal rat); y-axis: liver tissue from DEN- treated rat at 12^th ^week (A); at 14^th ^week (B); at 16^th ^week (C); at 20^th ^week (D). The red points represent 'present' states both in control and DEN exposed; blue points represent 'no present' in either of control and DEN-exposed; yellow points represent 'absent' states both in control and DEN-exposed.

Liver tissues were taken from the control and the DEN-treated rats. Total RNA was isolated from theses samples and used to prepare cRNA probes for hybridization with Affymetrix GeneChip Rat 230 2.0 arrays (Figure 3). The hybridized microarrays were then scanned and the signals acquired (Figure 4). At the 12^th ^week, liver cirrhosis occurred in 10 of 10 rats, so we took the pooled cirrhotic tissues from the 10 rats for the microarrays. At the 14^th ^week, dysplastic nodules occurred only in the livers of 2/10 rats, so we took the pooled dysplastic nodules from the two rats for the microarrays. At the 16^th ^week, early tumor nodules occurred in the liver of 8/10 rats, so we took the pooled tumor nodules from the eight rats for the microarrays. At the 20^th ^week, tumor nodules occurred in all of the ten rats(10/10), but lung metastasis only occurred in the two of them, so we took the pooled tumor nodules in the liver from the two rats with lung metastasis for the microarrays. We used the pooled liver tissues from the control rats killed at the 12^th^, 14^th^, 16^th ^and the 20^th ^week for the microarrays. The decision to pool the mRNA from the rat livers was made in order to obtain a representative analysis of gene expression changes across more than one animal.

### Analysis of the differential expression genes

The differential expression genes of cirrhotic tissue, dysplastic nodules, early tumors nodules and tumor nodules from rats with lung metastasis compared with the tissue from normal rats were screened and to determine the upregulated and downregulated DEGs. The results are shown in Table 1.

**Table 1:** Number of differential expression genes (DEGs) of liver tissues from DEN-treated rats compared with control.

DEGs	12^th ^week	14^th ^week	16^th ^week	20^th ^week
Up-regulated DEGs	681	857	1223	999
Down-regulated DEGs	687	732	1016	906
Total	1368	1589	2239	1905

NOTE: The words '12^th ^week, 14^th ^week, 16^th ^week, 20^th ^week' in the table indicate the cirrhosis tissue, dysplastic nodules, early cancerous nodules and cancerous nodules with metastasis, respectively.

The upregulated and the downregulated DEGs of cirrhosis tissue, dysplastic nodules, early tumor nodules and tumor nodules with lung metastasis vs the normal livers respectively were grouped into functional categories using gene ontology (GO) terms (Table 2). The GO terms such as metabolism, transport, cellular proliferation, apoptosis, adhesion, angiogenesis, etc. were chosen. Meanwhile, some other genes were associated with oxidative stress, immune response and inflammatory response.

**Table 2:** The deregulated DEGs sharing from cirrhosis to metastasis stage classified by the following screened GO.

Functional Categories	Number Of Annotated Genes				
	
	12^th ^week	14^th ^week	16^th ^week	20^th ^week	4 group
Metabolism	334/318	403/324	541/446	494/375	206/198
Transport	162/164	188/167	264/225	229/195	101/106
Cell Growth	129/88	161/86	207/104	218/88	89/51
Cell Differentiation	103/57	127/67	170/69	171/69	72/35
Apoptosis	87/50	113/48	128/62	153/46	59/28
Angiogenesis	12/11	15/13	23/15	25/14	9/6
Cell Proliferation	68/51	93/57	108/57	115/54	46/36
Cell Migration	13/12	15/15	30/13	25/13	10/8
Cell Adhesion	62/25	76/30	106/30	94/30	40/13
Extracellular Matrix	41/21	48/22	61/29	73/23	26/14
Oxidative Stress	31/19	41/24	43/27	50/26	23/12
Immune Response	30/25	34/23	38/35	35/28	19/16
Inflammatory Response	12/17	18/20	17/31	18/21	7/11
Cytochrome	19/30	23/28	29/45	25/38	11/20
Signal Transduction	140/106	165/111	243/129	213/115	87/59
Protein Kinase	114/67	128/77	193/95	185/73	65/38
Proteasome	17/6	20/8	25/7	19/6	13/4

NOTE: The words '12^th ^week, 14^th ^week, 16^th ^week, 20^th ^week' in the table indicate the cirrhosis tissue, dysplastic nodules, early cancerous nodules and cancerous nodules with metastasis, respectively. The word '4 group' means the DEGs sharing for the above 4 stages of liver tissues. The numbers up and down the line indicate the number of up-regulated and down-regulated DEGs respectively.

The histological changes during the hepatocarnogenesis in DEN-treated rat models were similar to those seen in humans, including non-specific damage, fibrosis, cirrhosis, dysplastic nodules, early tumorous nodules, progression and metastasis, which appeared to be sequential events. The processes of chronic inflammation, fibrosis and cirrhosis are closely related to liver cancer, while cirrhosis was considered as the precancerous lesions. Therefore, the co-expression of deregulated genes among these four stages might suggest they play key roles in the development of hepatocellular carcinoma. Among upregulated DEGs sharing from cirrhosis to metastasis, there were 246 known genes, 39 translocation loci, 51 inferred genes and 13 unkown genes; while among downregulated DEGs sharing from cirrhosis to metastasis, there were 215 known genes, 48 translocation loci, 63 inferred genes and 19 unkown genes (see additional file 1).

Cellular proliferation, apoptosis, adhesion, migration and agiogenesis all play important roles in carcinogenesis. Extracellular matrix (ECM) is important for the growth and progression of hepatocarcinogenesis (see additional file 2 and Tables 3). There are 105 upregulated and 51 downregulated DEGs with the above functions.

**Table 3:** The down-regulated DEGs sharing from cirrhosis to metastasis sorted out by the following GO function.

Gene Symbol	Gene Title	GO
COL18A1	procollagen, type XVIII, alpha 1	1–6
CXCL12	chemokine (C-X-C motif) ligand 12	1,2,4,5
KDR	kinase insert domain protein receptor	1,4,6
SERPINA3K	serine (or cysteine) peptidase inhibitor, clade A, member 3K	1,2,5
ANG1	angiogenin, ribonuclease A family, member 1	1,5
RNASE4	ribonuclease, RNase A family 4	1,5
C5	complement component 5	2,4
CML4	Camello-like 4	3
ENPP2	ectonucleotide pyrophosphatase/phosphodiesterase 2	3
GPHN	gephyrin	3
IGFALS	insulin-like growth factor binding protein, acid labile subunit	3
LIN7A	lin-7 homolog a (C. elegans)	3
AZGP1	alpha-2-glycoprotein 1, zinc	3,5
PROC	Protein C	2
PTPRD	protein tyrosine phosphatase, receptor type, D	3
PVRL3_predicted	poliovirus receptor-related 3 (predicted)	3
SORL1	sortilin-related receptor, LDLR class A repeats-containing	4,5
TGFBI	transforming growth factor, beta induced	3,4,6
RB1	retinoblastoma 1	2,3,5
EGFR	epidermal growth factor receptor	2–6
EGF	epidermal growth factor	2,5,6
IGF1	Insulin-like growth factor 1	2,5,6
HNF4A	Hepatocyte nuclear factor 4, alpha	2,5
BCL6_PREDICTED	B-cell leukemia 6 (predicted)	2,5
PEMT	phosphatidylethanolamine N-methyltransferase	2,5
LRP1	low density lipoprotein receptor-related protein 1	2,5
RGN	regucalcin	2,5
SGPP1	sphingosine-1-phosphate phosphatase 1	2
NR1D2	nuclear receptor subfamily 1, group D, member 2	2
GHR	Growth hormone receptor	2
CYP2E1	cytochrome P450, family 2, subfamily e, polypeptide 1	2
C4BPB	complement component 4 binding protein, beta	2
C6	complement component 6	2
FAAH	fatty acid amide hydrolase	2
NR0B2	nuclear receptor subfamily 0, group B, member 2	2
PCSK9	proprotein convertase subtilisin/kexin type 9	2
UNG	uracil-DNA glycosylase	2
CEBPA	CCAAT/enhancer binding protein (C/EBP), alpha	5
PCAF	p300/CBP-associated factor	5
CFB	complement factor B	5
DBP	D site albumin promoter binding protein	5
ADRA1B	adrenergic receptor, alpha 1b	5
FABP1	fatty acid binding protein 1, liver	5
VIPR1	vasoactive intestinal peptide receptor 1	5
ID4	Inhibitor of DNA binding 4	5
NOX4	NADPH oxidase 4	5
AMY1	amylase 1, salivary	6
GPLD1	glycosylphosphatidylinositol specific phospholipase D1	6
SMOC1	SPARC-related modular calcium binding protein 1	6

NOTE: The numbers from 1–6 indicate GO terms: angiogenesis, apoptosis, cell adhesion, cell migration, cell proliferation and extracellular matrix, respectively.

The rat models of liver cancer induced by DEN occurred following chronic injury, regenetation, fiborsis and cirrhosis. Elements of the inflammatory response, immune response and oxidative stress were also involved in the process of hepatocarcinogenesis. Tables 4 and 5 show that the expression of 40 such genes was upregulated and the expression of 27 genes was downregulated.

**Table 4:** The up-regulated DEGs sharing from cirrhosis to metastasis stage relating to the following GO process.

Gene symbol	Gene name	GO
CCL21B	chemokine (C-C motif) ligand 21b (serine)	1–2
CD276	CD276 antigen	1–2
SPP1	secreted phosphoprotein 1	1–2
CD24	CD24 antigen	1
C1QG	complement component 1, q subcomponent, gamma polypeptide	1
CD74	CD74 antigen	1
HLA-DMA	major histocompatibility complex, class II, DM alpha	1
HLA-DMB	major histocompatibility complex, class II, DM beta	1
DEFB1	defensin beta 1	1
FCGR3	Fc receptor, IgG, low affinity III	1
PLSCR1	phospholipid scramblase 1	1
PRNP	prion protein	1
RT1-BA	RT1 class II, locus Ba	1
RT1-CE5	RT1 class I, CE5	1
RT1-DA	RT1 class II, locus Da	1
RT1-DB1	RT1 class II, locus Db1	1
RT1-BB	RT1 class II, locus Bb	1
ANXA1	annexin A1	2
FABP4	fatty acid binding protein 4, adipocyte	2
S100A8	S100 calcium binding protein A8	2
S100A9	S100 calcium binding protein A9	2
CDC2A	cell division cycle 2 homolog A	3
EGR1	early growth response 1	3
CRYAB	crystallin, alpha B	3
CCND1	cyclin D1	3
CD36	cd36 antigen	3
GCLC	glutamate-cysteine ligase, catalytic subunit	3
GGT1	gamma-glutamyltransferase 1	3
GPX2	glutathione peroxidase 2	3
GPX3	glutathione peroxidase 3	3
GSR	glutathione reductase	3
GSS	glutathione synthetase	3
HSPCB	heat shock 90 kDa protein 1, beta	3
LAMC1	laminin, gamma 1	3
MTAP2	microtubule-associated protein 2	3
NOL3	nucleolar protein 3 (apoptosis repressor with CARD domain)	3
NQO1	NAD(P)H dehydrogenase, quinone 1	3
PDLIM1	PDZ and LIM domain 1 (elfin)	3
SLC25A4	solute carrier family 25	3
TXNRD1	thioredoxin reductase 1	3

NOTE: The numbers from 1–3 indicate immune response, inflammatory response and oxidative stress, respectively.

**Table 5:** The down-regulated DEGs sharing from cirrhosis to metastasis stage relating to the following GO process.

Gene Symbol	Gene Title	GO
C5	complement component 5	1–2
IL4RA	interleukin 4 receptor, alpha	1–2
MBL2	mannose binding lectin 2 (protein C)	1–3
NOX4	NADPH oxidase 4	2–3
ATRN	Attractin	2–3
C1S	complement component 1, s subcomponent	1
C4BPB	complement component 4 binding protein, beta	1
AZGP1	alpha-2-glycoprotein 1, zinc	1
C6	complement component 6	1
CXCL12	chemokine (C-X-C motif) ligand 12	1
MX2	myxovirus (influenza virus) resistance 2	1
OAS1	2',5'-oligoadenylate synthetase 1, 40/46 kDa	1
RT1-S3	RT1 class Ib, locus S3	1
VIPR1	vasoactive intestinal peptide receptor 1	1
APOA2	apolipoprotein A-II	2
BCL6_predicted	B-cell leukemia/lymphoma 6 (predicted)	2
KLKB1	kallikrein B, plasma 1	2
PROC	protein C	2
PTGER3	Prostaglandin E receptor 3 (subtype EP3)	2
MEOX2	mesenchyme homeobox 2	3
CA3	carbonic anhydrase 3	3
ABCB11	ATP-binding cassette, sub-family B (MDR/TAP), member 11	3
ALAD	aminolevulinate, delta-, dehydratase	3
CYP2E1	cytochrome P450, family 2, subfamily e, polypeptide 1	3
EGFR	epidermal growth factor receptor	3
HAO1	hydroxyacid oxidase 1	3
HNF4A	Hepatocyte nuclear factor 4, alpha	3

NOTE: The numbers from 1–3 indicate immune reponse, inflammatory response and oxidative stress, respectively.

### Hierarchy cluster analysis of DEGs

**Figure 5:**
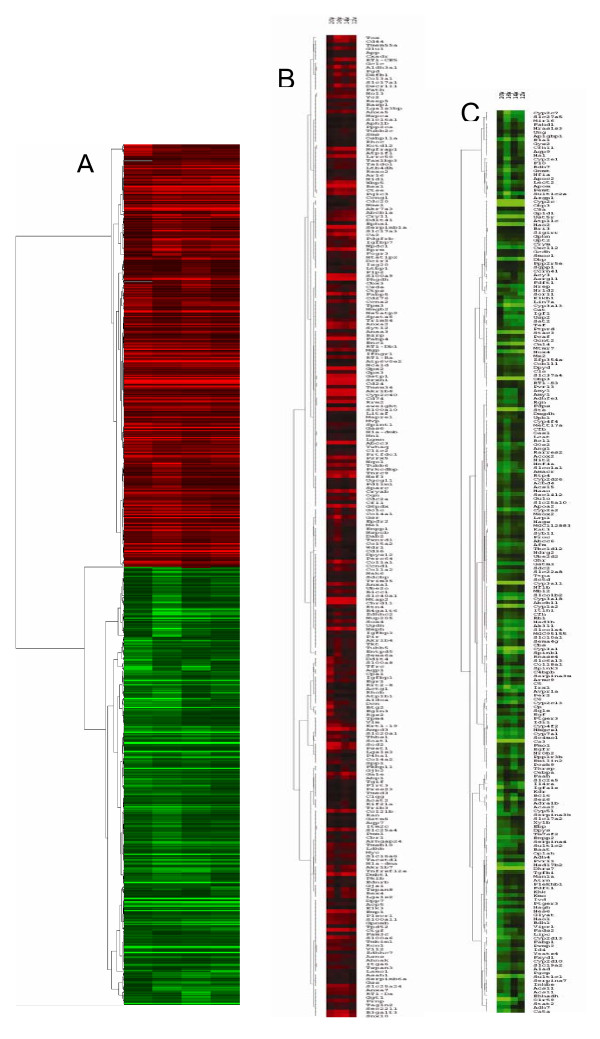
**Hierarchial clustering of screened differential expressional genes**. (A) hierarchical clustering of 694 deregulated genes shared in liver tissues of DEN-treated rats from cirrhosis tissues at the 12^th ^week, dysplastic nodules at the 14^th ^week, early cancerous nodules at the 16^th ^week, and cancerous nodules with lung metastasis at the 20^th ^week, respectively. (*Red*, a high expression level as compared with the mean; *green*, a low expression level as compared with the mean). (B) the dendrogram of the 246 upregulated known genes shared in the liver tissues of four chips is magnified. (C)the dendrogram of the 215 downregulated known genes shared in the liver tissues of four chips is magnified.

**Figure 6:**
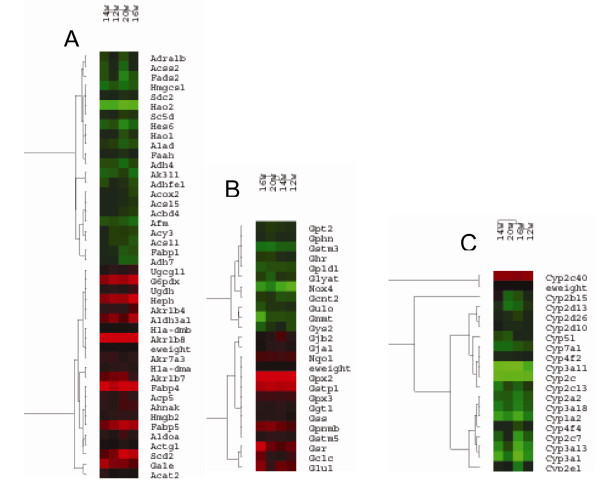
**Hierarchial clustering of deregulated genes involved in appointed functions**. (A) hierarchical clustering of deregulated genes involved in metabolism such as glucose, fat, alcohol and so on. (B) hierarchical clustering of 25 genes whose expression was significantly correlated with metabolism of glutathione. (C) all of the cytochrome P450 members deregulated shared in liver tissues of DEN-treated rats from the cirrhosis tissues at the 12^th ^week to the metastasis phase at the 20^th ^week.

The DEGs commonly present from stages of cirrhosis to metastasis were clustered by Cluster software according to the values of log_2 _ratio(Figure 5A,B,C). The upregulated (red) and the downregulated (green) DEGs had the same alterated tendency during the process from liver cirrhosis to metastasis. Furthermore, the DEGs involved in the metabolism of glucose, lipids and alcohol and so on (Figure 6A), DEGs associated with the metabolism of glutathione (Figure 6B) and DEGs of members belong to the CYPs family were listed (Figure 6C).

### Validation of differential expression of genes by real-time RT-PCR

**Figure 7:**
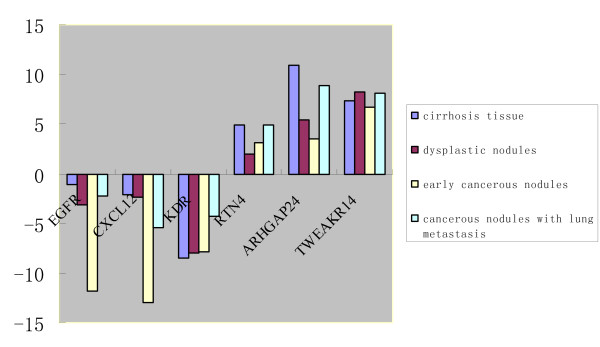
**Histogram of Ntarget value of the genes for validation by real time RT-PCR**. Each collumn represents Ntarget value of the corresponding target gene in cirrhosis tissues at the 12^th ^week, dysplastic nodules at the 14^th ^week, early cancerous nodules at the 16^th ^week and cancerous nodules with lung metastasis at the 20^th ^week.

The DEGs detected through Affymetrix genechip analysis were confirmed in the selected tissue of DEN-treated and control rats by real-time RT-PCR, as shown in Figure 7. TWEAKR, ANXA2, CTGF were chosen from the upregulated DEGs, and EGFR, KDR, CXCL12 were chosen from the downregulated DEGs. The primer sequences for each gene were listed in Table 6. The quality and specificity of the amplified products were confirmed by visualization on a 2% agarose gel. The results confirmed the validity of the Affymetrix genechip results. The lower the ΔCt value of the target gene, the more mRNA content of the target gene there is in the tissue. The Ct value of β-actin obtained from DEN-treated and normal tissue was almost identical.

**Table 6:** Primer sequences.

Primer name	Sequence	Product size (bp)
TWEAKR forward	5' GTGTTGGGATTCGGGTTGGTG 3'	221
TWEAKR reverse	5' CTAAGAGCGCCTCCCAGAATGG3'	
ARHGAP24 forward	5' TAGCATCAACTCCTTTCATCCC3'	232
ARHGAP24 reverse	5' TCCTTGACAAGGTTTGCCTG3'	
RTN4 forward	5' TGGTGGTTCTGTGCGGTGTG3'	248
RTN4 reverse	5' AGCTGTCCTTCACAGGTTC3'	
KDR forward	5'TTTCTGCTCCTTGGTCCTGG3'	222
KDR reverse	5' TGTCGCCACACTCAGTCAC 3'	
EGFR forward	5' CCTTAGCCGTCCTGTCCAAC 3'	235
EGFR reverse	5' TTGGGACAGCTCGGATCAC 3'	
CXCL12 forward	5' CCCTGCCGATTCTTTGAG3'	175
CXCL12 reverse	5' TGTTTAAGGCTTTGTCCAGG3'	
Beta-actin forward	5' AGGGTGTGATGGTGGGTATGGG 3'	230
Beta-actin reverse	5' TTCACGGTTGGCCTTAGGGTTC 3'	

## Discussion

Some major drawbacks limit the usefulness of studies regarding molecular pathogenesis in HCC. First, few studies analyze the genetic and genomic alterations that emerge at different time points during the entire progressive process of the disease. Second, the limited size of the studies is often a factor that undermines the capability to provide consistent genomic data[9]. Animal models of hepatocarcinogenesis summarize the primal biology of liver tumorigenesis and have provided reliable data for understanding the cellular development of HCC in humans[1,10,11]. In the present study, the pathologic changes of livers in rats treated by DEN included non-specific injuries, regeneration and repair, fibrosis, and cirrhosis, dysplastic nodules, early tumorous nodules, advanced tumorous nodules and metastasis foci, resembling the process of human hepatocarcinogenesis. DEGs obtained by compare normal rats with DEN-treated animals at stages from cirrhosis to metastasis allowed us to screen for upregulated and downregulated gene expressional profiles. The number of DEGs at each stage was large and the information obtained was powerful. We were thus able to visualize the complicated process of hepatocarcinogenesis at the genomic level.

The annotated information of the DEGs show that extensive and diverse biological processes and molecular functions are involved in hepatocaricnogenesis. Most of the DEGs are involved in metabolism and transport, indicating that significant alterations occurred in the process of metabolism and transport during the developmnet of HCC. For example, tumor cells always perform anaerobic glycolysis, even when there is an adequate oxygen supply[12,13], partly a result of alterations in the profile of enzymes associated with glycolysis. In this study, the gene expression level of lactate dehydrogenase B increased from the cirrhosis phase to the metastasis phase. Evidence shows that some genetic changes promoting tumor growth influences glucose energy metabolism directly[14,15]. Many intermediate products from glycolysis are used to synthesize proteins, nucleic acids and lipids by tumor cells, providing the essential materials for the growth and hyperplasia of tumor cells. For aggressive tumors, increased glycolysis and metabolism alterations often occurred. The microenvironment acidosis provided by the conversion of pyruvic acid to lactic acid promotes invasion and metastasis of tumor cells [16-18]. Therapy targeting glycolysis could preferentially kill tumor cells with metabolic alterations and there is experimental evidence that some types of tumors can be treated by inhibiting the glycolytic and phosphopentose pathways[16]. In this study, some DEGs associated with metabolisms of glucose were shown in Figure 6A.

Fat metabolism have significant changes in the process of tumorigenesis, e.g. a high fat diet was related to the development of many tumors [19]. Enhanced fat synthesis in tumor cells could not only support the increased membrane synthesis and energy metabolism, but also higher level of fatty acid synthetase provides the base for interpretation the relation between the fat metabolism and the capacity of hyperplasia and metastasis of tumor cells[20]. Stearoyl-CoA desaturase (SCD), which have four known isomers, takes part in regulating lipid synthesis. SCD2 plays key roles in the early development and survival of embryos in mice, whose expressional levels in the livers of wild mice embryos and newborn mice were higher than that of adult mice[21]. Inhibition of lipid synthesis caused by the depletion of SCD2 was related to the decreased expression level of peroxisome proliferator-activated receptor gamma (PPAR-γ)[22]. Fatty acid binding proteins (FABPs) are proteins that could bind to fatty acid and other lipids reversibly. Researchers found expression of FABP5, coding epidermal fatty acid binding protein (E-FABP-GenBank Accession), upregulated in primary tongue carcinomas[23]. FABP4, as a bridge between the inflammation and other metabolism syndromes[24], could not only transport the nuclear receptor PPAR-γ from cytoplasm to nucleus but also cause increased transcript activation of it[25]. In this study, the expressional levels of SCD2, FABP4 and FABP5 increased during the process from cirrhosis to metastasis in rat model, suggesting that an alteration of the fat metabolism occurred in hepatocarcinogenesis of rat model. Other DEGs associated with fatty metabolisms were shown in Figure 6A.

In the present study, some enzymes related to the glutathione (GSH) metabolism were found to be significantly altered. For example, the expressional level of Gstm3 (glutathione S-transferase, mu type 3) decreased in all stages of hepatocarcinogenesis, while the expression levels of of enzymes increased, which including Glul (Glutamate-ammonia ligase), Gclc (Glutamate-cysteine ligase, catalytic subunit), GPX2 (Glutathione peroxidase 2), GPX3 (Glutathioneperoxidase 3), GSR (Glutathione reductase), Yc2 (Glutathione S-transferase Yc2 subunit), Gstm5 (Glutathione S-transferase, mu 5), Gstp1 (Glutathione-S-transferase, pi 1) and GSS (Glutathione synthetase). Some studies reported that GSH and the associated enzymes were considered to promot the tumor transformation from dysplastic nodules and take part in the development and progression of hepatocarcinomas[26,27]. In gastroenteric tumors, the increase of GSH and the associated enzymes exhibit activation, indicating the existence of ROS and anti-oxidation defense related to GSH metabolism[28]. There were five binding sites for β-catenin/TCF at the promoter region of GPX2, indicating that GPX2 might take part in the corresponding signal pathways[29]. Thus previous research and our data indicate that genes related to oxidative stress and GSH metabolism play important roles in the process of progression from dysplastic nodules to tumor. The expressional level of GSH increased in tissue of HCC and the active hyperplasia liver cells[30,31]. Research has shown that DNA oxidative injury is increased in human HCC [32,33].

Many other enzymes associated with metabolism are involved in the defense and stress reaction, such as oxidative stress. For example, AKR1B7 (aldo-keto reductase family 1, member 7) takes part in the detoxification of oxides, such as aldehyde. During the detoxification of aldehyde, the expressional level of AKR1B7 mRNA increased. There are five binding sites with NF-κB at the 5' upstream region of the AKR1B7 gene, and oxidative stress upregulates the expression of AKR1B7 mediated by NF-κB[34,35]. The expression level of aldehyde dehydrogenase ALDH3A1 (Aldehyde dehydrogenase 3A1) also increased after oxidative stress. In the present study, the expression levels of AKR1B7, AKR1B8 and ALDH3A1 were up-regulated at all stages of hepatocarcinogenesis. In the tumor cells, reactive oxigen species (ROS) was produced through the oxidative stress. ROS as signal molecules mediate various reactions relating to growth, such as angiogenesis. ROS in endothelial cells is mainly from NADPH oxidation enzymes, consisting of Nox1, Nox2, Nox4, Nox5, p22 (phox), p47 (phox) and Rac1 (small G-protein Rac1). NADPH oxidative enzymes were activated by different factors including VEGF, angiopoietin-1, hypoxia and ischemia. Furthermore, ROS has been shown to be involved in spontaneous phosphorylation[36]. Nox4 mediated growth factors, such as anti-apoptosis of IGF, is partly due to the ROS produced by NADPH oxidative enzymes inhibiting the key protein tyrosine phosphatases(PTPs), then continually causing JAK2 kinase phosphorylation which resists the apoptosis reaction[37]. In this study, However, the gene expression level of NADPH oxidative enzymes decreased in the livers of our rat model at all stages of hepatocarcinogenesis. The mechanism is unclear.

Cytochrome P450s (CYPs) are key enzymes in tumorigenesis, taking part in the activation and inactivation of chemotherapeutic agents in tumor tissues[38]. The expression level of CYP1B1 in breast carcinomas was up-regulated significantly, providing a new therapy target and phenotype biomarker. The significant increase in CYP2E1 correlates with invasiveness and is a potential prognosis factor[39,40]. Other studies have shown that the expression of CYP could influence the synthesis of arachidonic acid derivatives, thus altering the various downstream signal pathways, which was thought to be the prelude of carcinogenesis[41]. At present, it is not possible to always predict the efficacy of a chemotherapeutic agent or its degree of toxicity. Analysis of CYPs expressional levels in tumor cells may allow prognosis decisions and therapy predictions. In this study, only the expression level of CYP2C40 increased at all stages of hepatocarcinogenesis in rat models, while the remaining CYPs decreased (Figure 6C). Clearly, further investigation is needed to determine the role(s) of CYPs in hepatocarcinogenesis.

In addition to the deregulated expression of metabolism associated genes, we noticed that among the DEGs in the hepatocarcinogenesis of rat models, some known tumor-associated genes, such as Rb1 and Myc, showed deregulated expression occurring at all the stages of hepatocarcinogenesis. Their persisting activation or deactivation could induce the tumor phenotype, as well as play roles at the later stage of progression and metastasis. Meanwhile, some known metastasis-associated genes are found deregulated at the promotion stage of tumor development. For example, the expression level of Ndrg2 and Hrasls3 (HRAS like suppressor 3) decreased at all stages compared to the normal livers, while the expression level of Nme1 (expressed in non-metastatic cells 1) increased. Generally, it was thought that genes involved in the development of carcinoma activation participated at the early stage, while genes participating in the metastasis were activated at the latter stage of tumor progression[42]. In opposition to the traditional model, Bernards and Weinberg proposed that the metastatic ability of tumor cells occurred at the early stage of tumor development[43]. Some oncogenes such as Ras and Src assigned the tumor cells with the metastatic phenotype [44-46].

As we known, the important characteristic of malignant tumor cells is the capability of invading the vicinity, forming metastasis foci at the remote organ, overcoming the host's control over the microenvironment[47,48]. The malignant transformation of liver cells occurred on the basis of chronic injury, regeneration and cirrhosis. The liver cancer cells could synthesize ECM components and the ECM surrounding liver cancer cells was found to be different from that of stroma in the normal organ [49-51]. Integrin and laminin are the major components of ECM. The interaction between integrin and laminin is closely related to the signal transduction, providing survival signals for the cells, mediating the liver cancer cells formation of pseudopodia, and adherence with laminin, which are imperative if a liver cancer cell is to migrate and invade [52-55]. In the process of hepatocarcinogenesis in this rat model, the deregulated expression of many ECM associated genes plays important roles in the hepatocarcinogenesis, e.g. Itga6, Lamc1, Col1a1 and Spp1, etc. (Table 2, 3 and additonal file 2). The differential expression profile of ECM associated genes in time course and space is very important to cellular proliferation and migration.

It is worthy of note that the expression of the annexin family members and calcium-binding proteins are deregulated in this rat model of liver cancer. In our study, the expressional level of Annexin A1, A2, A3, A5 and A7 increased compared with the normal liver tissue. Annexins consist of a conserved protein family. Annexin A2 is closely associated with cell division regulation and tumor growth, and is deregulated in many tumors[56,57]. Two Annexin A2 molecules bind to the long chains of p11/S100A10 dimers through its N-terminals, form the isotetramer, regulating the reactions of Annexin A2 and membranes and actin in cortical areas, and the distribution of recirculating endosomes[58]. In addition, S100A10 and Annexin A2 form isodimers, prompting the invasion and metastasis of the tumor by activating plasminogen[59]. In the present study, the expression level of S100a10, S100a11, S100a6, S100a8 and S100a9 increased from cirrhosis to metastatic process when compared with the normal liver. S100A8/A9 form the compounds that play a role in inducing apoptosis in tumor cells. S100A8/A9 at low concentrations prompts growth activity, the phosphorylation of MAPK pathway and NF-κB is activated in cells after S100A8/A9 treatment.

The majority of HCCs slowly unfold against a background of chronic hepatitis and cirrhosis, which can be considered as preneoplastic conditions of the liver. Chronic hepatitis is characterized by persistent inflammation, cytokine and oxidative stress-mediated hepatocyte death and active proliferation of residual hepatocytes to replace the lost parenchyma[1,60]. During the process of hepatocarcinogenesis in rat models, chronic inflammation precedes cirrhosis. Epidemiology studies showed that chronic inflammation increased the risk of tumors, and the microenvironment of tumorigenesis resembles the reaction of inflammation to injury in many ways[61]. In the tumor microenvironment, the chemotactic factors and receptors mediated angiogenesis, recruited cells, prompting cellular survival and proliferation. On the other hand, oxidative stress occurred in inflammatory processes. The inflammatory cells and tumor cells both produce free radicals and soluble factors such as arachidonic acid, cytokines and chemotactic factors, seubsequently producing reactive oxygen. All these factors strongly recruit the inflammatory cells to produce cytokines, which promotes a vicious cycle. The intermediate products of active oxygen oxidize DNA directly or interfere with DNA repair. These oxides activate protein, carbohydrate and lipids quickly, the derived products interfere with inter- and intracellular homeostasis, favoring DNA mutation. Thus, the chronic inflammation prompts the malignant transformation of cells[62]. Chronic inflammation also favors angiogenesis[63]. In the present study, many DEGs are related to inflammation reaction, immune reaction and stress. (Tables 4 and 5)

## Conclusion

In conclusion, analysis of gene expression profile is a useful tool to provide new clues and produce new research targets in the hepatocarcinogenesis field. In the present study, a rat model of liver cancer was established. We have listed the deregulated expression genes in the process from cirrhosis to liver cancer in the DEN-treated rat model. As indicated in the literature, this model shows that cirrhosis is a precancerous lesion of the liver. Although we only discuss some parts of the great quantity of information in this study, much unknown information remains. Functional analysis of these genes revealed discrete expression clusters, including cell proliferation, protein synthesis, and hepatocyte-specific functions. We still need to discern the key genes playing core roles in the promotion and progression of liver cancer. In this article, we focused our attention on the global molecular events that occurred in DEN-treated rats (and probably represent the earliest ones that start the multistep process of hepatocarcinogenesis). Additional information may be mined from this and similar studies to provide clues to many areas including the very important search for diagnostic markers, therapy targets and prognosis prediction markers.

## Abbreviations

HCC: Hepatocellular carcinoma; DEN: diethylnitrosamine; DEGs: differential expression genes.

## Competing interests

The authors declare that they have no competing interests.

## Authors' contributions

YFL wrote the manuscript. BSZ performed the validation of genes. HLZ and XJZ established the animal model. YHL prepared the tissue slides. JZ helped write the manuscript. JPZ, ZQF and XHG participated in the design of the study and helped to draft the manuscript. All authors read and approved the final manuscript.

## Supplementary Material

Additional file 1 **The list of deregulated DEGs sharing from cirrhosis to metastasis stage compared with control**. A table for all the screened DEGs sharing from stage of liver cirrhosis to metastasis. Click here for file

Additional file 2 **The up-regulated DEGs sharing from cirrhosis to metastasis sorted out by the following GO function**. for the screened DEGs sharing from stage of liver cirrhosis to metastasis sorted out by the GO words: angiogenesis, apoptosis, cell adhesion, cell migration, cell proliferation and extracellular matrix. Click here for file
