# Supplementary material for: Characteristic gene expression profiles in the progression from liver cirrhosis to carcinoma induced by diethylnitrosamine in a rat model
Source: J Exp Clin Cancer Res. 2009 Jul 29;28(1):107. doi: 10.1186/1756-9966-28-107 (PMC2729293; doi:10.1186/1756-9966-28-107)
Supplement: Additional file 1 — The list of deregulated DEGs sharing from cirrhosis to metastasis stage compared with control. A table for all the screened DEGs sharing from stage of liver cirrhosis to metastasis. [file 1756-9966-28-107-S1.pdf]

—

Table 1 The list of deregulated DEGs sharing from cirrhosis to metastasis stage compared with control.

Probe Set Id	Gene Title	Gene Symbol	Log_2 ratio			
			12w	14w	16w	20w
1367811_At	3-Phosphoglycerate Dehydrogenase	Phgdh	5.3	5.1	5.7	6.6
1376226_At	Acetyl-Coenzyme A Acetyltransferase 2	Acat2	1	1.9	1.1	1.2
1367942_At	Acid Phosphatase 5, Tartrate Resistant	Acp5	1.7	2	1.9	2.3
1371327_A_At	Actin, Gamma, Cytoplasmic 1	Actg1	1	1.1	1.1	1.6
1368342_At	Adenosine Monophosphate Deaminase 3	Ampd3	3.4	4.3	5.2	5.4
1371703_At	Ahnak Nucleoprotein (Desmoyokin)	Ahnak	1.7	1.9	2.3	2.4
1368130_At	Aldehyde Dehydrogenase Family 3, Member A1	Aldh3a1	3.7	3	4.5	2.7
1367734_At	Aldo-Keto Reductase Family 1, Member B4 (Aldose Reductase)	Akr1b4	2	1.9	2.6	1.9
1368569_At	Aldo-Keto Reductase Family 1, Member B7	Akr1b7	3.3	2.9	2	3.4
1370902_At	Aldo-Keto Reductase Family 1, Member B8	Akr1b8	8.8	8.9	8.7	8.5
1368121_At	Aldo-Keto Reductase Family 7, Member A3 (Aflatoxin Aldehyde Reductase)	Akr7a3	2.1	2	1.9	1.4
1367617_At	Aldolase A	Aldoa	1.3	1.8	1.7	2.3
1368413_At	Amiloride Binding Protein 1 (Amine Oxidase, Copper-Containing)	Abp1	2.5	3.2	1.9	2.6
1371571_At	Amyloid Beta (A4) Precursor Protein	App	1.6	1.7	1.7	1

1367614_At	Annexin A1	Anxa1	2.9	3.1	3.9	3.5
1367584_At	Annexin A2	Anxa2	3.6	3.2	3.7	4.2
1367974_At	Annexin A3	Anxa3	1.3	1.5	1.5	1.5
1386862_At	Annexin A5	Anxa5	2.9	3.3	2.7	2.9
1368143_At	Annexin A7	Anxa7	2.5	3	3	2.8
1388440_At	Anterior Pharynx Defective 1b Homolog	Aph1b	1.4	1.6	1.2	1.2
1369625_At	Aquaporin 1	Aqp1	2.1	1.9	1.7	3.7
1368317_At	Aquaporin 7	Aqp7	1.1	1.3	1.1	1.5
1387925_At	Asparagine Synthetase	Asns	2	2.2	2.6	2.5
1369588_A_At	Atpase Inhibitory Factor 1	Atpif1	2.5	2.6	2.5	2
1372907_At	Atpase, H+ Transporting, V0 Subunit E Isoform 2	Atp6v0e2	1	1.2	1	1.1
1367814_At	Atpase, Na+/K+ Transporting, Beta 1 Polypeptide	Atp1b1	1	1.1	1	1.8
1370583_S_At	Atp-Binding Cassette, Sub-Family B (Mdr/Tap), Member 1b	Abcb1a	2.9	2.9	2.5	2
1369698_At	Atp-Binding Cassette, Sub-Family C (Cftr/Mrp), Member 3	Abcc3	4.5	4.8	5.3	4.8
1386995_At	B-Cell Translocation Gene 2, Anti-Proliferative	Btg2	1.8	1.6	2.1	4.7
1370249_At	Benzodiazepine Receptor, Peripheral	Bzrp	2.2	2.7	2.6	2.7
1398350_At	Brain Abundant, Membrane Attached	Basp1	2.1	2.4	2.2	2

—

	Signal Protein 1					
1388802_At	Brain Expressed X-Linked 1	Bex1	8.2	8.6	9.2	7
1373458_At	Brain Expressed X-Linked 4	Bex4	2.7	2.8	1.7	2.8
	Candidate Mediator Of The P53-Dependent					
1390672_At	G2 Arrest	Rprm	4.7	4.3	3.9	4.1
1386922_At	Carbonic Anhydrase 2	Ca2	4.4	4.1	4.5	3.4
1368037_At	Carbonyl Reductase 1	Cbr1	1.9	2.3	1.6	2.3
1369657_At	Carboxypeptidase A1	Cpa1	2.4	2.2	2.2	4.1
1368167_At	Cathepsin E	Ctse	3.1	3.3	3.5	2.7
1369953_A_At	Cd24 Antigen	Cd24	4.6	4.6	4.5	4.8
1374198_At	Cd276 Antigen	Cd276	1.2	1	1.4	1.4
1367689_A_At	Cd36 Antigen	Cd36	1	1.6	2	1.1
1387952_A_At	Cd44 Antigen	Cd44	2.9	3.9	3.8	2.4
1367679_At	Cd74 Antigen	Cd74	2.8	2.8	2.8	2.8
1367776_At	Cell Division Cycle 2 Homolog A	Cdc2a	3	2.4	2.8	2.7
1370294_A_At	Cell Division Cycle 20 Homolo	Cdc20	2	1.4	1.3	1.6
1374557_At	Cg6210-Like	Loc362065	1.2	1.7	2.1	1.8
	Chemokine (C-C Motif) Ligand 21b					
1378015_At	(Serine)	Ccl21b	3.6	4	3.2	4.3
1381993_At	Chloride Intracellular Channel 2	Clic2	1.7	1.7	1.8	1.6
	Chromobox Homolog 3 (Hp1 Gamma					
1371395_At	Homolog, Drosophila)	Cbx3	1.1	1	1.3	1.5

—

1370184_At	Cofilin 1, Non-Muscle	Cfl1	1.5	1.3	1.6	1.5
1370376_A_At	Cold Shock Domain Protein A	Csda	2	1.9	2.4	2.7
1373025_At	Complement Component 1, Q Subcomponent, Gamma Polypeptide	C1qg	1	1.3	1	1
1367631_At	Connective Tissue Growth Factor	Ctgf	3.8	4.6	4.7	5.1
1384816_At	Coxsackie Virus And Adenovirus Receptor	Cxadr	1.7	1.8	1.7	1.1
1370026_At	Crystallin, Alpha B	Cryab	4.5	3.5	4.3	3.6
1376051_At	Crystallin, Lamda 1	Cryl1	3.2	3	2.7	2.3
1379582_A_At	Cyclin A2	Ccna2	3.3	2.6	3.2	3.2
1371150_At	Cyclin D1	Ccnd1	1.7	2.3	2.7	1.8
1367764_At	Cyclin G1	Ccng1	1.4	2.1	1.6	1.1
1368155_At	Cytochrome P450, Family 2, Subfamily C, Polypeptide 40	Cyp2c40	4.7	4.7	4.5	4.7
1370956_At	Decorin	Dcn	1.7	2.5	2.3	3.4
1369660_At	Defensin Beta 1	Defb1	3.4	2.8	3.7	1.9
1368007_At	Deleted In Malignant Brain Tumors 1	Dmbt1	4.4	4.9	3.5	5.5
1389553_At	Dendritic Cell Inhibitory Receptor 3	Dcir3	1.6	1.7	1.4	1.6
1371694_At	Dihydropyrimidinase-Like 2	Dpysl2	1.3	1.5	2.1	1.5
1367722_At	Dipeptidylpeptidase 7	Dpp7	1.1	1.4	1.3	1.6
1368202_A_At	Disabled Homolog 2 (Drosophila)	Dab2	1.6	1.5	2.7	1.8
1368025_At	Dna-Damage-Inducible Transcript 4	Ddit4	1.6	1.9	2.4	3.5
1368013_At	Dna-Damage-Inducible Transcript 4-Like	Ddit4l	3.5	3.2	3.2	2.6

—

	Down Syndrome Critical Region Gene					
1389066_At	1-Like 1	Dscr1l1	3.5	3.3	4.3	2.2
1368321_At	Early Growth Response 1	Egr1	2.2	2.7	2.2	3.6
1388666_At	Ectodermal-Neural Cortex 1	Enc1	1.7	2.1	1.9	2
	Ectonucleoside Triphosphate					
1382434_At	Diphosphohydrolase 5	Entpd5	1.7	1.4	2.5	1
	Ectonucleotide					
1370047_At	Pyrophosphatase/Phosphodiesterase 1	Enpp1	1.2	1.3	2.2	1.7
1368174_At	Egl Nine Homolog 3 (C. Elegans)	Egln3	1.2	1.5	1.6	2.9
1387146_A_At	Endothelin Receptor Type B	Ednrb	1.9	1.8	1.4	2.5
1375855_At	Ependymin Related Protein 2 (Zebrafish)	Epdr2	1.9	2	3	1.8
1371527_At	Epithelial Membrane Protein 1	Emp1	3.6	4.1	4.1	4.6
1387669_A_At	Epoxide Hydrolase 1, Microsomal	Ephx1	1.4	1.2	1.2	1
1370295_At	Expressed In Non-Metastatic Cells 1	Nme1	1.6	1.1	1	1.1
	Family With Sequence Similarity 3,					
1393412_At	Member C	Fam3c	1.2	1.5	1.5	1.8
1367654_At	Fat Tumor Suppressor Homolog	Fath	2.1	2.1	2.6	1.4
1368271_A_At	Fatty Acid Binding Protein 4, Adipocyte	Fabp4	4.6	5.4	5	5.5
1370281_At	Fatty Acid Binding Protein 5, Epidermal	Fabp5	3.9	3.2	4.2	4.6
1398246_S_At	Fc Receptor, Igg, Low Affinity Iii	Fcgr3	2.1	2.2	1.9	2
1372653_At	Fk506 Binding Protein 11	Fkbp11	1.2	2.1	1	1.5
1368074_At	Galactose-4-Epimerase, Udp	Gale	3.5	4.3	2.7	3.4

1368374_A_At	Gamma-Glutamyltransferase 1	Ggt1	1.5	1.8	1.7	1.5
1372002_At	Gap Junction Membrane Channel Protein Alpha 1	Gja1	1.3	1.8	1	1.7
1373386_At	Gap Junction Membrane Channel Protein Beta 2	Gjb2	1.7	2.4	1.2	1.5
1367856_At	Glucose-6-Phosphate Dehydrogenase X-Linked	G6pdx	4.4	3.7	4.8	4.1
1389426_At	Glutamate-Ammonia Ligase (Glutamine Synthase)	Glul	2.6	3	3.5	2
1370688_At	Glutamate-Cysteine Ligase, Catalytic Subunit	Gclc	2.1	1.5	3	1.8
1374070_At	Glutathione Peroxidase 2	Gpx2	7	6.7	7	7.2
1369926_At	Glutathione Peroxidase 3	Gpx3	2.2	2.1	2.1	2.2
1369061_At	Glutathione Reductase	Gsr	3	2.5	4.9	3
1371089_At	Glutathione S-Transferase Yc2 Subunit	Yc2	3.2	3.4	3	2.9
1370813_At	Glutathione S-Transferase, Mu 5	Gstm5	1.5	1.8	1.5	1.9
1370365_At	Glutathione Synthetase	Gss	1.2	1.7	1.6	1.6
1388122_At	Glutathione-S-Transferase, Pi 1	Gstp1	4.4	4.3	4.2	4.5
1368187_At	Glycoprotein (Transmembrane) Nmb	Gpnmb	2.4	2.9	3.2	3.4
1383047_At	Growth Arrest Specific 6	Gas6	1.7	1.7	2	1.9
1375335_At	Heat Shock 90kda Protein 1, Beta	Hspcb	1.3	1.3	2.3	1.9
1388850_At	Heat Shock Protein 1, Alpha	Hspca	1.5	1.7	1.5	1.5

—

1371336_At	Hematological And Neurological Expressed Sequence 1	Hn1	1.7	1.7	1.9	1.7
1368533_At	Hephaestin	Heph	4.2	3.7	5.3	4.1
1367676_At	High Mobility Group Box 2	Hmgb2	1.9	1.5	1.9	2
1393050_At	Hypothetical Loc302495	Loc302495	1.2	1.2	2	1.2
1383422_At	Hypothetical Loc362564	Loc362564	4.5	3.9	4.3	3.7
1373088_At	Hypothetical Protein Loc682888	Loc682888	2.7	3	2.2	1.9
1368160_At	Insulin-Like Growth Factor Binding Protein 1	Igfbp1	1.5	2.1	1.6	3
1367652_At	Insulin-Like Growth Factor Binding Protein 3	Igfbp3	1.9	1.8	2.3	1.9
1371357_At	Insulin-Like Growth Factor Binding Protein 7	Igfbp7	2.3	2.2	1.9	2.2
1371334_At	Integral Membrane Protein 2c	Itm2c	1.1	1.3	1	1.3
1383240_At	Integrin, Alpha 6	Itga6	1.4	1.7	1.9	1.8
1369956_At	Interferon Gamma Receptor 1	Ifngr1	1	1.1	1	1.1
1390507_At	Interferon Stimulated Exonuclease 20	Isg20	1.3	1.4	1	1.2
1388433_At	Keratin Complex 1, Acidic, Gene 19	Krt1-19	2.2	2.5	3.5	3.5
1371530_At	Keratin Complex 2, Basic, Gene 8	Krt2-8	1.1	1.4	1.1	1.8
1376193_At	Kohjirin	Chrdl1	4.6	5.1	5.7	4.9
1370218_At	Lactate Dehydrogenase B	Ldhb	2.2	2.2	2.1	2.9
1371322_At	Laminin, Gamma 1	Lamc1	1.2	1.5	1.8	1.7

	Latent Transforming Growth Factor Beta					
1367912_At	Binding Protein 1	Ltbp1	2.2	2.2	1.6	1.8
1386879_At	Lectin, Galactose Binding, Soluble 3	Lgals3	1.9	1.8	2.5	2.8
1368278_At	Lectin, Galactoside-Binding, Soluble 2	Lgals2	2.7	3	1.5	2.8
	Lectin, Galactoside-Binding, Soluble, 3					
1387946_At	Binding Protein	Lgals3bp	1.7	1.9	1.7	1.5
1368430_At	Legumain	Lgmn	1.7	1.8	1.9	1.7
1382932_At	Leucine Rich Repeat Containing 50	Lrrc50	4.1	4.1	4.2	3.5
1388102_At	Leukotriene B4 12-Hydroxydehydrogenase	Ltb4dh	2.2	2.3	2.4	1.7
1370928_At	Lps-Induced Tn Factor	Litaf	1.5	1.5	1.6	1.7
	Major Histocompatibility Complex, Class					
1370904_At	Ii, Dm Alpha	Hla-Dma	1.8	1.6	1.5	1.9
1367925_At	Major Vault Protein	Mvp	1	1.1	1.2	1.2
1370870_At	Malic Enzyme 1	Me1	1.9	1.8	2.7	1.6
1367568_A_At	Matrix Gla Protein	Mgp	3.1	3.5	3	3.5
1388152_At	Microtubule-Associated Protein 2	Mtap2	4.6	5.3	6.3	5
	Microtubule-Associated Protein, Rp/Eb					
1373397_At	Family, Member 1	Mapre1	1.1	1.1	1.2	1.3
	Myelocytomatosis Viral Oncogene					
1368308_At	Homolog (Avian)	Myc	2	2	1.8	2.5
1374770_At	N-Acylsphingosine Amidohydrolase 1	Asah1	1.1	1.8	1.5	1.8
1387599_A_At	Nad(P)H Dehydrogenase, Quinone 1	Nqo1	2.3	2.2	2.4	2.3

1369948_At	Nerve Growth Factor Receptor (Tnfrsf16) Associated Protein 1	Ngfrap1	4.3	4.2	4.3	3.3
1372773_At	Neural Proliferation, Differentiation And Control, 1	Npdc1	2.5	2.3	2.1	2.4
1372953_At	Neurocalcin Delta	Ncald	1.5	1.4	1.4	1.6
1367845_At	Neurofilament 3, Medium	Nef3	5.3	3.4	4.9	3.9
1371518_At	Nidogen 1	Nid1	1.9	2.1	2.3	1.7
1374565_At	Nima (Never In Mitosis Gene A)-Related Expressed Kinase 6	Nek6	1.5	1.6	1.9	1.7
1388340_At	Ns5a (Hepatitis C Virus) Transactivated Protein 9	Ns5atp9	2.3	1.7	1.9	2.3
1368544_A_At	Nucleolar Protein 3 (Apoptosis Repressor With Card Domain)	Nol3	2.5	2.6	2.3	2.2
1383633_At	Oxysterol Binding Protein-Like 1a	Osbpl1a	1.3	1.7	1.5	1.2
1367946_At	Pdz And Lim Domain 1 (Elfin)	Pdlim1	1.8	1.3	1.6	1.6
1372294_At	Pe Responsive Protein C64	Perc64	1	1.2	1.6	1.1
1371646_At	Phosphogluconate Dehydrogenase (Mapped)	Pgd	2.2	1.8	2.7	1.6
1369029_At	Phospholipid Scramblase 1	Plscr1	2.3	2.6	2.7	3
1371455_At	Phosphomannomutase 1	Pmm1	1.4	1.6	1.2	1.6
1372665_At	Phosphoserine Aminotransferase 1	Psat1	3.5	2.8	4	4.9
1377662_At	Pirin	Pir	1.5	1.4	1.8	1.4

—

	Platelet Derived Growth Factor Receptor,					
1370642_S_At	Beta Polypeptide	Pdgfrb	3.1	2.3	2.5	2.2
1390839_At	Pq Loop Repeat Containing 3	Pqlc3	3.5	3.7	3.9	3.1
1370156_At	Prion Protein	Prnp	2.4	3.1	3.1	2.7
1388116_At	Procollagen, Type 1, Alpha 1	Col1a1	3.2	3.3	4.7	3.2
1370155_At	Procollagen, Type I, Alpha 2	Col1a2	2.2	2.6	3.3	2.2
1370959_At	Procollagen, Type Iii, Alpha 1	Col3a1	2.2	1.9	2.5	1.5
1372439_At	Procollagen, Type Iv, Alpha 1	Col4a1	1.9	1.5	2.8	1.9
1370895_At	Procollagen, Type V, Alpha 2	Col5a2	1.3	1.3	2	1.5
	Procollagen-Proline, 2-Oxoglutarate					
	4-Dioxygenase (Proline 4-Hydroxylase),					
1370954_At	Alpha 1 Polypeptide	P4ha1	1	1	1.3	1.6
1373152_At	Protease, Serine, 23	Prss23	1	1.5	1.1	1.2
1367691_At	Protein Kinase C, Delta Binding Protein	Prkcdbp	2.3	2.2	2.2	2.1
	Protein Kinase Inhibitor Beta, Camp					
1369105_A_At	Dependent, Catalytic	Pkib	1.5	1.6	1.1	1.9
	Protein Phosphatase 2 (Formerly 2a),					
1398790_At	Catalytic Subunit, Alpha Isoform	Ppp2ca	1.2	1.4	1.1	1
1388393_At	Proteolipid Protein 2	Plp2	1.2	1.2	1.3	1.6
	Purinergic Receptor P2x, Ligand-Gated Ion					
1369674_At	Channel, 5	P2rx5	3.7	3.9	4.1	3.4
1367590_At	Ran, Member Ras Oncogene Family	Ran	1.1	1.2	1	1.3

1369958_At	Ras Homolog Gene Family, Member B	Rhob	1.5	1.7	1.8	2.6
1368144_At	Regulator Of G-Protein Signaling 2	Rgs2	1.2	1.9	2	3.7
1368888_A_At	Reticulon 4	Rtn4	1.8	2.1	2.3	1.9
1371724_At	Rex2, Rna Exonuclease 2 Homolog	Rexo2	1.5	1.6	1.6	1.1
1392590_At	Rho Gtpase Activating Protein 24	Arhgap24	1.4	1.4	1.2	1.6
1389408_At	Ribonucleotide Reductase M2	Rrm2	4.1	4.2	4.2	4.2
1371209_At	Rt1 Class I, Ce5	Rt1-Ce5	3.7	4	3.8	1.9
1377334_At	Rt1 Class Ii, Locus Ba	Rt1-Ba	2.9	3.2	2.8	3.1
1371065_At	Rt1 Class Ii, Locus Bb	Rt1-Bb	2.3	3	2.4	2.7
1370883_At	Rt1 Class Ii, Locus Da	Rt1-Da	2.4	2.8	2.7	2.6
1370383_S_At	Rt1 Class Ii, Locus Db1	Rt1-Db1	1.8	2.3	2.1	2.1
1386890_At	S100 Calcium Binding Protein A10 (Calpactin)	S100a10	3.8	4.2	4	4
1375170_At	S100 Calcium Binding Protein A11 (Calizzarin)	S100a11	2.5	2.7	2.7	3.1
1367661_At	S100 Calcium Binding Protein A6 (Calcyclin)	S100a6	2.7	3.5	3.6	4.2
1368494_At	S100 Calcium Binding Protein A8 (Calgranulin A)	S100a8	2	2.2	2.6	3.8
1387125_At	S100 Calcium Binding Protein A9 (Calgranulin B)	S100a9	4.1	4.3	4.7	5.5
1377691_At	Sec22 Vesicle Trafficking Protein-Like 1	Sec22l1	1.6	1.9	2	1.6

1367562_At	Secreted Acidic Cysteine Rich Glycoprotein	Sparc	1.6	1.1	1.5	1.4
1367581_A_At	Secreted Phosphoprotein 1	Spp1	2.1	1.8	2.9	2.7
1386981_At	Solute Carrier Family 16 (Monocarboxylic Acid Transporters), Member 1	Slc16a1	1	1.2	1	1
1378301_At	Solute Carrier Family 16 (Monocarboxylic Acid Transporters), Member 6	Slc16a6	2.7	2.6	2.2	2.7
1387188_At	Solute Carrier Family 17 (Sodium Phosphate), Member 1	Slc17a1	2.2	1.8	2.3	1.4
1390591_At	Solute Carrier Family 17 (Sodium Phosphate), Member 3	Slc17a3	3.2	3	3.1	2.6
1370314_At	Solute Carrier Family 20 (Phosphate Transporter), Member 1	Slc20a1	1.4	1.8	2.3	2.3
1388112_At	Solute Carrier Family 25 (Mitochondrial Carrier; Adenine Nucleotide Translocator), Member 4	Slc25a4	3.2	3.7	2.9	3.8
1387130_At	Solute Carrier Family 39 (Iron-Regulated Transporter), Member 1	Slc40a1	1.6	1.7	2.1	1.6
1383585_S_At	Sorting Nexin 10	Snx10	3.2	4.9	4.3	3.6
1368643_At	Spermatogenesis Associated 6	Spata6	1.7	1.3	1.4	1.6
1375889_At	Spermine Synthase	Sms	1.5	2	1.6	1.4
1367668_A_At	Stearoyl-Coenzyme A Desaturase 2	Scd2	3.4	2.7	4.6	5.2
1374976_A_At	Sterol O-Acyltransferase 1	Soat1	1	1.8	2.3	2.5

—

1372510_At	Sulfiredoxin 1 Homolog (S. Cerevisiae)	Srxn1	5.8	5.7	5.6	6.1
1398762_At	Syndecan Binding Protein	Sdcbp	1.2	1.3	1.5	1.4
1388392_At	Tax1 (Human T-Cell Leukemia Virus Type I) Binding Protein 3	Tax1bp3	2.1	2.1	2.1	1.8
1371361_At	Tensin	Tns	1.4	1.6	1.5	1.7
1398325_At	Tetraspanin 3	Tspan3	1.9	2.3	2.6	2.4
1368052_At	Tetraspanin 8	Tspan8	2.4	3.2	1.7	3.5
1373421_At	Tg Interacting Factor	Tgif	1.5	1.9	1.2	1.6
1386958_At	Thioredoxin Reductase 1	Txnrd1	1.8	1.7	3.2	2.1
1374529_At	Thrombospondin 1	Thbs1	1.9	2.6	3.4	3
1367655_At	Thymosin, Beta 10	Tmsb10	2.7	2.6	2.2	3.2
1369940_At	Transaldolase 1	Taldo1	2.2	2.3	2.2	1.9
1388750_At	Transferrin Receptor	Tfrc	1.9	1.5	2	3
1388335_At	Transgelin 2	Tagln2	1.7	2.1	2.2	1.9
1386859_At	Transketolase	Tkt	2.1	1.9	2.7	1.9
1376102_At	Transmembrane Bax Inhibitor Motif Containing 1	Tmbim1	2.3	2.3	2.8	2.8
1388628_At	Transmembrane Emp24 Domain Containing 3	Tmed3	1.2	1.7	1.2	1.3
1379419_At	Transmembrane Protein 34	Tmem34	2.2	2.2	2.2	2.3
1383375_At	Transmembrane Protein 55a	Tmem55a	1.6	1.9	1.6	1
1370694_At	Tribbles Homolog 3 (Drosophila)	Trib3	1.1	2	1.1	2.2

—

1371786_At	Tripartite Motif Protein 35	Trim35	1.3	1.5	1.7	1.5
1372639_At	Tripartite Motif-Containing 54	Trim54	2.5	2.2	2.3	2.9
1398303_S_At	Tropomyosin 3, Gamma	Tpm3	1.8	1.4	1.7	1.8
1368838_At	Tropomyosin 4	Tpm4	1.4	1.5	2	2
1367579_A_At	Tubulin, Alpha 6	Tuba6	1.7	1.5	1.6	1.9
1371390_At	Tubulin, Beta 2c	Tubb2c	1.8	2.1	1.7	1.4
1387892_At	Tubulin, Beta 5	Tubb5	2	1.9	2.6	1.8
1376100_At	Tubulin, Beta 6	Tubb6	3.7	3.3	3.8	3.3
1371785_At	Tumor Necrosis Factor Receptor Superfamily, Member 12a	Tnfrsf12a	2.8	2.7	1.7	3.1
1388199_At	Tumor-Associated Calcium Signal Transducer 1	Tacstd1	2.2	2.1	1.7	2.2
1370168_At	Tyrosine 3-Monooxygenase	Ywhaq	1.4	1.4	1.4	1.4
1398373_At	Udp-Gal:Betaglcnac Beta 1,3-Galactosyltransferase, Polypeptide 3	B3galt3	2.7	4.2	4.2	3.5
1387206_At	Udp-Gal:Betaglcnac Beta 1,4-Galactosyltransferase, Polypeptide 6	B4galt6	1.3	1.2	1.6	1
1368953_At	Udp-Glucose Ceramide Glucosyltransferase-Like 1	Ugcgl1	1.9	1.3	2.1	1.7
1367938_At	Udp-Glucose Dehydrogenase	Ugdh	1.9	1.7	2.2	1.6
1370615_At	Udp-Glucuronosyltransferase	Loc286989	7.7	7.3	7.9	6.3
1370875_At	Villin 2	Vil2	2.4	2.6	3	3

1367574_At	Vimentin	Vim	1.8	2	2.7	2.7
1389520_At	Wd Repeat Domain 1	Wdr1	1	1	1.6	1.2
1370828_At	Zinc Finger, Dhhc Domain Containing 2	Zdhhc2	2.3	2.2	3	1.8
1368152_At	Zinc Finger, Dhhc Domain Containing 7	Zdhhc7	1.2	1.3	1.5	1.5
1375003_At	Serine (Or Cysteine) Peptidase Inhibitor, Clade B, Member 6a	Serpinb6a	1	1.6	1.4	1.5
1377034_At	Serine (Or Cysteine) Proteinase Inhibitor, Clade B, Member 1a	Serpinb1a	6	5.4	5.8	5
1380346_At	Serine (Or Cysteine) Proteinase Inhibitor, Clade B, Member 1a	Serpinb1a	5.2	4.2	4.8	3.6
1383290_At	Serine Peptidase Inhibitor, Kunitz Type 1	Spint1	2	2.2	2.3	2.3
Genes is inferred and predicted (51)						
Translocation locus (39)						
Unknown completely (13)						
1371152_A_At	2',5'-Oligoadenylate Synthetase 1, 40/46kda	Oas1	-1.3	-1.3	-1.9	-1.2
1367932_At	3-Hydroxy-3-Methylglutaryl-Coenzyme A Synthase 1	Hmgcs1	-2.2	-2.8	-2.3	-2.8
1368794_At	3-Hydroxyanthranilate 3,4-Dioxygenase	Haao	-1.4	-1.4	-2	-1.4
1368387_At	3-Hydroxybutyrate Dehydrogenase, Type 1	Bdh1	-1.2	-1.2	-1.8	-2.6
1368091_At	5-Oxoprolinase (Atp-Hydrolysing)	Oplah	-1	-1.4	-1.4	-1.7
1388153_At	Acyl-Coa Synthetase Long-Chain Family Member 1	Acsl1	-1.8	-1.3	-2.3	-1.8

	Acyl-Coa Synthetase Long-Chain Family					
1386926_At	Member 5	Acsl5	-1.1	-1.1	-1.6	-1.4
	Acyl-Coenzyme A Binding Domain					
1373253_At	Containing 4	Acbd4	-1.2	-1.2	-1.8	-1.6
	Acyl-Coenzyme A Oxidase 2, Branched					
1371137_At	Chain	Acox2	-1.2	-1.2	-1.9	-1.4
1371824_At	Adenylate Kinase 3-Like 1	Ak3l1	-2.3	-2.5	-2.8	-1.8
1368574_At	Adrenergic Receptor, Alpha 1b	Adra1b	-1	-1.8	-1.3	-2
1371266_At	Afamin	Afm	-2	-2.2	-2.8	-2.3
	Alcohol Dehydrogenase 4 (Class Ii), Pi					
1369863_At	Polypeptide	Adh4	-2	-2	-1.9	-2.7
	Alcohol Dehydrogenase 7 (Class Iv), Mu					
1369072_At	Or Sigma Polypeptide	Adh7	-1.1	-1.2	-2.4	-2.5
1389548_At	Alcohol dehydrogenase, iron containing, 1	Adhfe1	-1.8	-1.7	-2.6	-1.8
1387234_At	Alpha-2-Glycoprotein 1, Zinc	Azgp1	-1.2	-1.7	-3.1	-1.5
1370425_At	Alpha-2u Globulin Pgcl3	Loc259244	-9.7	-9.8	-9.8	-8.8
1387985_A_At	Alpha-2u Globulin Pgcl4	Obp3	-9.2	-7.8	-8.9	-7.1
1388031_X_At	Alpha-2u Globulin Pgcl5	Loc259245	-7.9	-7.5	-7.9	-10
1367775_At	Alpha-Methylacyl-Coa Racemase	Amacr	-2.3	-1.9	-3.3	-2.6
1367720_At	Aminolevulinate, Delta-, Dehydratase	Alad	-1.8	-1.6	-2.1	-2.4
1370359_At	Amylase 1, Salivary	Amy1	-1.2	-2.3	-2.3	-1.7
1390326_At	Angiogenin, Ribonuclease A Family,	Ang1	-1.5	-1.8	-2.6	-1.9

	Member 1					
1388450_At	Ap1 Gamma Subunit Binding Protein 1	Ap1gbp1	-1.8	-1.2	-2.2	-1.2
1369727_At	Apolipoprotein A-Ii	Apoa2	-1.4	-1.5	-1.9	-1.4
1393139_At	Apolipoprotein C-Ii	Apoc2	-1.4	-1	-2.8	-1.2
1386980_At	Apolipoprotein M	Apom	-1.5	-1.6	-2.8	-1.6
1368621_At	Aquaporin 9	Aqp9	-1.6	-1	-2.2	-1.3
1369664_At	Arginine Vasopressin Receptor 1a	Avpr1a	-2.5	-3.5	-3	-1.9
1374871_At	Asparaginase Like 1	Asrgl1	-1.8	-1.2	-1.6	-1.5
1389785_At	Aspartoacylase (Aminoacylase) 3	Acy3	-1.8	-1.1	-1.9	-1.7
1368769_At	Atp-Binding Cassette, Sub-Family B (Mdr/Tap), Member 11	Abcb11	-1.3	-1.5	-1.7	-1.4
1368452_At	Atp-Binding Cassette, Sub-Family C (Cftr/Mrp), Member 6	Abcc6	-1.4	-1.6	-1.9	-1.7
1388038_At	Attractin	Atrn	-1.1	-1.4	-1.2	-1.7
1387508_At	Bile Acid-Coenzyme A: Amino Acid N-Acyltransferase	Baat	-1.2	-1.8	-1.7	-2.1
1371833_At	Brain Protein I3	Bri3	-1	-1	-1.1	-1.1
1387336_At	Camello-Like 4	Cml4	-4.6	-4.6	-3.9	-3.6
1367896_At	Carbonic Anhydrase 3	Ca3	-5.5	-6.9	-5.9	-6.7
1387609_At	Carbonic Anhydrase 5a, Mitochondrial	Ca5a	-1.5	-1.8	-3.7	-3.1
1384127_At	Ccaat/Enhancer Binding Protein (C/Ebp), Alpha	Cebpa	-1.8	-1.4	-1.1	-1.8

—

1377869_At	Ccr4 Carbon Catabolite Repression 4-Like	Ccrn4l	-1.4	-1.1	-1.1	-1.4
1368418_A_At	Ceruloplasmin	Cp	-1.1	-1.5	-1.2	-1
1388583_At	Chemokine (C-X-C Motif) Ligand 12	Cxcl12	-3.1	-2.6	-3.2	-3.2
1369852_At	Coagulation Factor X	F10	-1.1	-1	-2.1	-1
1387893_At	Complement Component 1, S Subcomponent	C1s	-1.4	-1.3	-1.5	-1.2
1368695_At	Complement Component 4 Binding Protein, Beta	C4bpb	-1.5	-1.7	-2.4	-1.2
1383425_At	Complement Component 5	C5	-1.7	-1.6	-2.4	-1.3
1384580_At	Complement Component 6	C6	-2.2	-2.8	-2.6	-1.8
1370676_At	Complement Component Factor H	Cfh	-1.4	-1.7	-1.9	-1.4
1382678_At	Complement Component Factor H-Like 1	Cfhl1	-1.8	-1.2	-2.3	-1.6
1389470_At	Complement Factor B	Cfb	-1.1	-1.1	-1.7	-1
1368059_At	Crystallin, Mu	Crym	-1.9	-1.7	-2	-2.2
1387178_A_At	Cystathionine Beta Synthase	Cbs	-1.3	-1.8	-2	-1.2
1370495_S_At	Cytochrome P450 2c13	Cyp2c13	-2	-3.8	-3.1	-2.4
1398307_At	Cytochrome P450, 3a18	Cyp3a18	-3	-3.8	-3.9	-3
1387243_At	Cytochrome P450, Family 1, Subfamily A, Polypeptide 2	Cyp1a2	-3.8	-4.6	-5.1	-4.1
1371076_At	Cytochrome P450, Family 2, Subfamily B, Polypeptide 15	Cyp2b15	-1.6	1.7	-2.6	-3.2
1370241_At	Cytochrome P450, Family 2, Subfamily C,	Cyp2c7	-2.9	-3	-4.4	-1.5

	Polypeptide 7					
1370496_At	Cytochrome P450, Family 2, Subfamily D, Polypeptide 13	Cyp2d13	-1.9	-1.8	-3.2	-3.6
1367917_At	Cytochrome P450, Family 2, Subfamily D, Polypeptide 26	Cyp2d26	-1.4	-1.3	-2.2	-1.8
1370377_At	Cytochrome P450, Family 2, Subfamily D, Polypeptide 9	Cyp2d10	-1.4	-1.1	-1.7	-1.7
1367871_At	Cytochrome P450, Family 2, Subfamily E, Polypeptide 1	Cyp2e1	-1.3	-1.4	-3.7	-1.1
1387118_At	Cytochrome P450, Family 3, Subfamily A, Polypeptide 1	Cyp3a1	-4.2	-5.1	-6	-3.6
1370593_At	Cytochrome P450, Family 3, Subfamily A, Polypeptide 11	Cyp3a11	-5.1	-6.4	-6.7	-6.7
1370387_At	Cytochrome P450, Family 3, Subfamily A, Polypeptide 13	Cyp3a13	-4	-4.1	-5.1	-2.4
1368467_At	Cytochrome P450, Family 4, Subfamily F, Polypeptide 2	Cyp4f2	-1.3	-1.7	-1.4	-1.7
1387973_At	Cytochrome P450, Family 4, Subfamily F, Polypeptide 4	Cyp4f4	-1.4	-1.5	-2.4	-1.5
1368458_At	Cytochrome P450, Family 7, Subfamily A, Polypeptide 1	Cyp7a1	-2.4	-3.3	-2.8	-3.3
1369424_At	Cytochrome P450, Subfamily 2a,	Cyp2a2	-2.8	-3	-3.8	-2.9

	Polypeptide 1					
1367979_S_At	Cytochrome P450, Subfamily 51	Cyp51	-1.6	-2.4	-1.5	-2.8
1387328_At	Cytochrome P450, Subfamily Iic	Cyp2c	-5.7	-6.1	-5.9	-5.9
1387874_At	D Site Albumin Promoter Binding Protein	Dbp	-4.3	-3.4	-3.3	-3.8
1397205_At	Dehydrogenase/Reductase (Sdr Family) Member 7	Dhrs7	-6	-5.8	-5.6	-7.5
1387284_At	Dihydropyrimidinase	Dpys	-1.4	-1.7	-2.3	-2.1
1367994_At	Dihydropyrimidine Dehydrogenase	Dpyd	-1.6	-1.5	-1.6	-1.3
1370936_At	Dimethylglycine Dehydrogenase Precursor	Dmgdh	-1.1	-1.4	-1.6	-1.3
1368536_At	Ectonucleotide Pyrophosphatase/Phosphodiesterase 2	Enpp2	-2.3	-2.6	-3.2	-3.1
1387819_At	Elastase 1, Pancreatic	Ela1	-3.5	-2	-4.1	-2.9
1368283_At	Enoyl-Coenzyme A, Hydratase/3-Hydroxyacyl Coenzyme A Dehydrogenase	Ehhadh	-2.3	-1.1	-2.6	-2.8
1368325_At	Epidermal Growth Factor	Egf	-1.2	-1.7	-1.4	-1.4
1370830_At	Epidermal Growth Factor Receptor	Egfr	-1.7	-2.4	-1.4	-1.7
1367667_At	Farensyl Diphosphate Synthase	Fdps	-1.3	-1.7	-1.8	-1.6
1389906_At	Farnesyl Diphosphate Farnesyl Transferase 1	Fdft1	-1.7	-1.4	-1	-1.2
1367839_At	Farnesyl Diphosphate Farnesyl Transferase 1	Fdft1	-1.4	-2	-1.6	-2.3

—

1368717_At	Fatty Acid Amide Hydrolase	Faah	-1.3	-1.2	-1	-1.5
1369111_At	Fatty Acid Binding Protein 1, Liver	Fabp1	-1.4	-1.3	-2.1	-2.3
1368453_At	Fatty Acid Desaturase 2	Fads2	-1.4	-2.1	-2.2	-3.2
1387053_At	Flavin Containing Monooxygenase 1	Fmo1	-1.6	-1.9	-1.6	-1.8
1372676_At	Fumarylacetoacetate Hydrolase Domain Containing 1	Fahd1	-1.7	-1.3	-2.2	-1.3
1369960_At	Fxyd Domain-Containing Ion Transport Regulator 1	Fxyd1	-1.2	-1.1	-1.6	-1.6
1388395_At	G0/G1 Switch Gene 2	G0s2	-2.2	-1.8	-3.1	-2.2
1368117_At	Gephyrin	Gphn	-1.3	-1.3	-1.3	-1.5
1374903_At	Glucosaminyl (N-Acetyl) Transferase 2, I-Branching Enzyme	Gcnt2	-2	-2.1	-1.6	-1.5
1387023_At	Glutathione S-Transferase, Mu Type 3	Gstm3	-2.3	-2.4	-2.7	-2.8
1387672_At	Glycine N-Methyltransferase	Gnmt	-1.9	-2	-4	-2
1380905_At	Glycine-N-Acyltransferase	Glyat	-1.6	-1.5	-1.7	-2.3
1368328_At	Glycogen Synthase 2	Gys2	-1.9	-1.1	-2.3	-1.5
1380833_At	Glycosylphosphatidylinositol Specific Phospholipase D1	Gpld1	-1.9	-2	-2.2	-2
1373803_A_At	Growth Hormone Receptor	Ghr	-1.3	-1.4	-1.6	-1.7
1374625_At	Hairy And Enhancer Of Split 6 (Drosophila)	Hes6	-2.1	-2.4	-2.6	-3.3
1391485_At	Hepatocyte Nuclear Factor 4, Alpha	Hnf4a	-1.5	-2	-2.8	-2.2

—

1387307_At	Histidine Ammonia Lyase	Hal	-1.7	-1.2	-2.4	-1.3
1370202_At	Hras Like Suppressor 3	Hrasls3	-1.7	-1.4	-2.1	-1.3
1394112_At	Hydroxyacid Oxidase 1	Hao1	-1.3	-1.3	-1.5	-2.1
1387139_At	Hydroxyacid Oxidase 2 (Long Chain)	Hao2	-4	-4.1	-4.4	-4.6
1369986_At	Hydroxyacyl Glutathione Hydrolase	Hagh	-1.1	-1.3	-1.4	-1.7
1387156_At	Hydroxysteroid (17-Beta) Dehydrogenase 2	Hsd17b2	-3.4	-3.1	-3.1	-4
1376496_At	Hypothetical Protein Loc503164	Loc503164	-3.9	-1.3	-1.5	-1.7
1398634_At	Hypothetical Protein Loc678836	Loc678836	-1.3	-1.5	-2.2	-2.1
1383692_At	Hypothetical Protein Loc681037	Loc681037	-1.8	-1.4	-2.2	-1.3
1369238_At	Inhibin Beta E	Inhbe	-1.6	-1.1	-2.1	-1.8
1394022_At	Inhibitor Of Dna Binding 4	Id4	-1.9	-1.6	-2.4	-2.6
1382599_At	Insulin-Like Growth Factor 1	Igf1	-2.2	-2.2	-2.5	-1.1
	Insulin-Like Growth Factor Binding					
1387816_At	Protein, Acid Labile Subunit	Igfals	-3.4	-2.1	-1.9	-4.4
1369450_At	Integral Membrane Transport Protein Ust5r	Ust5r	-4.3	-4.7	-4.7	-4.7
1368134_A_At	Interleukin 4 Receptor, Alpha	Il4ra	-3.5	-2.7	-1.6	-2.9
1368878_At	Isopentenyl-Diphosphate Delta Isomerase	Idi1	-1.4	-2.5	-1.8	-2.1
1370232_At	Isovaleryl Coenzyme A Dehydrogenase	Ivd	-1.1	-1.3	-1.5	-1.8
1387323_At	Kallikrein B, Plasma 1	Klkb1	-2	-1.9	-2.2	-1.1
1387375_At	Ketohexokinase	Khk	-1.1	-1.3	-1.3	-1.8
1367948_A_At	Kinase Insert Domain Protein Receptor	Kdr	-1.6	-1.1	-1.2	-2.2
1368915_At	Kynurenine 3-Monooxygenase (Kynurenine	Kmo	-1.7	-2.2	-2	-2.9

	3-Hydroxylase)					
1374006_At	Kynurenine Aminotransferase Iii	Kat3	-1.4	-1.5	-2	-1.7
1367887_At	Lecithin Cholesterol Acyltransferase	Lcat	-1.3	-1.2	-2.2	-1.5
1369837_At	L-Gulonolactone Oxidase	Gulo	-1.4	-1.5	-2.1	-1.5
1370108_A_At	Lin-7 Homolog A (C. Elegans)	Lin7a	-3.1	-2.9	-3.4	-1.5
1369701_At	Lipase, Hepatic	Lipc	-1.4	-1.7	-1.9	-3.1
1371715_At	Loc500651	Mgc112883	-1.6	-1.4	-2	-1.5
	Low Density Lipoprotein Receptor-Related					
1388416_At	Protein 1	Lrp1	-1.2	-1.1	-1.4	-1.1
1387528_At	Mannose Binding Lectin 2 (Protein C)	Mbl2	-1.2	-1.6	-1.6	-1.3
1398296_At	Membrane Interacting Protein Of Rgs16	Mir16	-2.4	-1.6	-3.2	-1
1368422_At	Mesenchyme Homeobox 2	Meox2	-1.7	-1.5	-1.9	-1.5
1371479_At	Methyltransferase Like 7a	Mettl7a	-1.2	-1.1	-1.9	-1.2
	Moco Sulphurase C-Terminal Domain					
1376847_At	Containing-Like	Loc690745	-1.9	-1.5	-2.2	-3.1
	Moco Sulphurase C-Terminal Domain					
1383796_At	Containing-Like	Loc690745	-1.8	-1.5	-1.9	-2.8
1387283_At	Myxovirus (Influenza Virus) Resistance 2	Mx2	-1.8	-1.7	-1.7	-1.3
1368543_At	Nadph Oxidase 4	Nox4	-4.3	-3.8	-3.4	-3
1371412_A_At	Neuronal Regeneration Related Protein	Nrep	-2.8	-2.6	-2	-2.2
1372438_At	Nitrilase Family, Member 2	Nit2	-1.3	-1.3	-2.1	-1.6
1387121_A_At	N-Myc Downstream Regulated Gene 2	Ndrg2	-1.5	-1.5	-1.8	-1.6

1369679_A_At	Nuclear Factor I/A	Nfia	-1.5	-1.3	-3	-1.7
1389601_At	Nuclear Factor I/B	Nfib	-1.6	-1.2	-1.8	-1.1
1368376_At	Nuclear Receptor Subfamily 0, Group B, Member 2	Nr0b2	-1.7	-2.5	-1.5	-1.7
1370541_At	Nuclear Receptor Subfamily 1, Group D, Member 2	Nr1d2	-2.6	-2	-1.5	-1.4
1387981_At	Olfactory Receptor 59	Olr59	-1.9	-3.3	-5.3	-5.4
1367729_At	Ornithine Aminotransferase	Oat	-2.4	-2.4	-3	-1.2
1375205_At	P300/Cbp-Associated Factor	Pcaf	-1.5	-1.6	-1.4	-1.4
1368303_At	Period Homolog 2 (Drosophila)	Per2	-1.2	-1.8	-1.6	-1
1367885_At	Peroxisomal Membrane Protein 2	Pxmp2	-1.2	-1.1	-1.9	-1.8
1386990_At	Phenylalkylamine Ca2+ Antagonist (Emopamil) Binding Protein	Ebp	-1.3	-1.4	-1.8	-1.9
1369581_At	Phosphatidylethanolamine N-Methyltransferase	Pemt	-1.2	-1.3	-2.3	-1.3
1368399_A_At	Plasma Glutamate Carboxypeptidase	Pgcp	-1.3	-1.2	-1.6	-1.7
1370334_At	Pleckstrin Homology Domain Containing, Family B (Evectins) Member 1	Plekhb1	-1.1	-1.4	-1.2	-1.7
1388459_At	Procollagen, Type Xviii, Alpha 1	Col18a1	-1.2	-1.3	-1.6	-1
1385640_At	Proprotein Convertase Subtilisin/Kexin Type 9	Pcsk9	-1.3	-2.3	-1.2	-1.4
1380389_At	Prostaglandin E Receptor 3 (Subtype Ep3)	Ptger3	-1.8	-2.6	-2.4	-2.3

—

1369286_At	Protein C	Proc	-1	-1.1	-1.5	-1.3
1384262_At	Protein Phosphatase 1, Regulatory (Inhibitor) Subunit 3b	Ppp1r3b	-2.2	-3.2	-2	-2.3
1387901_At	Protein Tyrosine Phosphatase, Receptor Type, D	Ptprd	-1.8	-1.8	-1.7	-1.5
1368627_At	Regucalcin	Rgn	-1.4	-2.3	-2.5	-1.9
1388185_At	Retinoblastoma 1	Rb1	-1.6	-1.8	-2	-1.5
1371691_At	Retinoic Acid Receptor Responder (Tazarotene Induced) 2	Rarres2	-1.3	-1.4	-2.1	-1.5
1387240_At	Retinol Dehydrogenase 7	Rdh7	-1.8	-1.4	-3.2	-1.5
1368943_At	Ribonuclease, Rnase A Family 4	Rnase4	-1.4	-2	-2.6	-1.3
1373452_At	Rna Terminal Phosphate Cyclase-Like 1	Rcl1	-1.1	-1.1	-2	-1.3
1388164_At	Rt1 Class Ib, Locus S3	Rt1-S3	-1.5	-1.2	-1.4	-1.1
1374308_At	Sec14-Like 2 (S. Cerevisiae)	Sec14l2	-2	-1.9	-2.8	-2
1391032_At	Seizure Related 6 Homolog (Mouse)	Sez6	-2	-3.8	-1.7	-4.6
1371143_At	Serine (Or Cysteine) Peptidase Inhibitor, Clade A (Alpha-1 Antipeptidase, Antitrypsin), Member 7	Serpina7	-1.9	-1.1	-2.5	-1.9
1368048_At	Serine (Or Cysteine) Peptidase Inhibitor, Clade A, Member 3k	Serpina3k	-1.7	-1.9	-2.8	-2.8
1370836_At	Serine (Or Cysteine) Proteinase Inhibitor, Clade A (Alpha-1 Antiproteinase,	Serpina4	-1.7	-1.9	-2.4	-2.2

—

	Antitrypsin), Member 4					
1371147_At	Serine (Or Cysteine) Proteinase Inhibitor, Clade A, Member 3m	Serpina3m	-2.2	-2.3	-3.3	-1.8
1371083_At	Serine Protease Inhibitor	Loc299282	-2.3	-2.8	-3.6	-3.2
1368446_At	Serine Protease Inhibitor, Kazal Type 1	Spink1	-2.9	-3.7	-4.4	-2.4
1387967_At	Serine Protease Inhibitor, Kazal Type 3	Spink3	-2.2	-2.2	-3	-2
1373670_At	Signal Transducer And Activator Of Transcription 2	Stat2	-1.1	-2.1	-3.2	-3
1378605_At	Single Immunoglobulin And Toll-Interleukin 1 Receptor (Tir) Domain	Sigirr	-1.1	-1.1	-1.2	-1.2
1368609_At	Solute Carrier Family 10 (Sodium/Bile Acid Cotransporter Family), Member 1	Slc10a1	-2.2	-2.8	-3.5	-2.2
1390863_At	Solute Carrier Family 19 (Thiamine Transporter), Member 2	Slc19a2	-2	-1.7	-2.3	-2.3
1368460_At	Solute Carrier Family 2, Member 5	Slc2a5	-1.5	-1.4	-1.1	-1.8
1368461_At	Solute Carrier Family 22 (Organic Anion Transporter), Member 8	Slc22a8	-1.8	-2	-2.3	-2.3
1370020_At	Solute Carrier Family 25 (Mitochondrial Carrier; Dicarboxylate Transporter), Member 10	Slc25a10	-1.3	-1.3	-1.9	-1.4
1387325_At	Solute Carrier Family 27 (Fatty Acid Transporter), Member 5	Slc27a5	-2.4	-2.4	-3.5	-1.4

	Solute Carrier Family 37 (Glycerol-6-Phosphate Transporter),					
1386960_At	Member 4	Slc37a4	-1.6	-1.6	-1.8	-1.3
	Solute Carrier Family 6 (Neurotransmitter					
1387372_At	Transporter, Gaba), Member 13	Slc6a13	-1.3	-2	-2.1	-1
	Solute Carrier Organic Anion Transporter					
1387567_At	Family, Member 1a1	Slco1a1	-1.6	-2.1	-3	-2.3
	Solute Carrier Organic Anion Transporter					
1387093_At	Family, Member 1a4	Slco1a4	-3.2	-3.7	-3.8	-2.7
	Solute Carrier Organic Anion Transporter					
1369746_A_At	Family, Member 1b2	Slco1b2	-2.2	-2.8	-2.9	-2.4
	Sortilin-Related Receptor, Ldlr Class A					
1377457_A_At	Repeats-Containing	Sorl1	-3.1	-1.7	-1.7	-1.8
	Sparc-Related Modular Calcium Binding					
1388545_At	Protein 1	Smoc1	-1.4	-1.2	-1.3	-1.3
1373874_At	Sphingosine-1-Phosphate Phosphatase 1	Sgpp1	-1.3	-1.1	-1.1	-1.1
1387017_At	Squalene Epoxidase	Sqle	-1.9	-2.5	-1.7	-1.6
1369465_At	Steroid Delta-Isomerase, 3 Beta	Hsd3b	-2.4	-2.9	-3.2	-2.1
1368275_At	Sterol-C4-Methyl Oxidase-Like	Sc4mol	-2.1	-2.7	-2.3	-2.9
	Sterol-C5-Desaturase (Fungal Erg3,					
1387926_At	Delta-5-Desaturase) Homolog	Sc5d	-1.2	-1.4	-1.6	-1.7
1369296_At	Sulfotransferase Family, Cytosolic, 1c,	Sult1c1	-3.8	-3.1	-4	-4.7

	Member 1					
	Sulfotransferase Family, Cytosolic, 1c,					
1369531_At	Member 2	Sult1c2	-1.3	-2.3	-2.4	-2.8
	Sulfotransferase Family, Cytosolic, 1c,					
1370943_At	Member 2a	Sult1c2a	-2.7	-2.8	-5	-2.2
1368733_At	Sulfotransferase, Estrogen Preferring	Ste	-3.9	-5.4	-5.5	-4.9
1370098_At	Synaptobrevin-Like 1	Sybl1	-1.3	-1.4	-1.9	-1.6
1370166_At	Syndecan 2	Sdc2	-1.2	-1.3	-1.4	-1.5
1387852_At	Thyroid Hormone Responsive Protein	Thrsp	-1.7	-3	-1.5	-2.1
1370150_A_At	Thyroid Hormone Responsive Protein	Thrsp	-1.6	-3.3	-1.5	-1.8
1385374_At	Thyrotroph Embryonic Factor	Tef	-2.4	-2.6	-2	-1.3
1369435_At	Tocopherol (Alpha) Transfer Protein	Ttpa	-1.3	-1.5	-1.7	-1.7
1371913_At	Transforming Growth Factor, Beta Induced	Tgfbi	-1.7	-1.6	-1.6	-2.1
1389725_At	Transmembrane 7 Superfamily Member 2	Tm7sf2	-1.3	-1.7	-2.1	-2
1387703_A_At	Ubiquitin Specific Peptidase 2	Usp2	-2.9	-3	-3	-1.2
1374890_At	Ubiquitin-Conjugating Enzyme E2d 2	Ube2d2	-1.1	-1.1	-1.3	-1.2
1389738_At	Uracil-Dna Glycosylase	Ung	-1.3	-1	-1.6	-1
1368245_At	Ureidopropionase, Beta	Upb1	-1	-1.6	-1.7	-1.6
1389270_X_At	Urinary Protein 2	Rup2	-1	-1.6	-1.6	-1.5
1383695_At	Vasoactive Intestinal Peptide Receptor 1	Vipr1	-1.1	-1	-1.2	-2.1
1390857_At	Xylulokinase Homolog (H. Influenzae)	Xylb	-1.2	-1.3	-1.8	-2
1368877_At	Zinc Finger Protein 354a	Zfp354a	-2.2	-2	-2	-1.7

—

Genes are inferred or predicted (63)

Transcribed Locus (48)

Unknown genes completely (19)

Note: The words ‘12w, 14w, 16w and 20w’ in the table indicate cirrhosis tissues, dysplastic nodules, early cancerous nodules and cancerous nodules with lung metastasis compared with control respectively. The signal log ratio estimates the magnitude and direction of change of a transcript when two arrays are compared (DEN-exposed versus control). The log scale used is base 2, thus, a Signal Log Ratio of 1.0 indicates an increase of the transcript level by 2 fold and -1.0 indicates a decrease by 2 fold.
