# Supplementary material for: Characteristic gene expression profiles in the progression from liver cirrhosis to carcinoma induced by diethylnitrosamine in a rat model
Source: J Exp Clin Cancer Res. 2009 Jul 29;28(1):107. doi: 10.1186/1756-9966-28-107 (PMC2729293; doi:10.1186/1756-9966-28-107)
Supplement: Additional file 2 — The up-regulated DEGs sharing from cirrhosis to metastasis sorted out by the following GO function. for the screened DEGs sharing from stage of liver cirrhosis to metastasis sorted out by the GO words: angiogenesis, apoptosis, cell adhesion, cell migration, cell proliferation and extracellular matrix. [file 1756-9966-28-107-S2.pdf]

Table 2. The up-regulated DEGs sharing from cirrhosis to metastasis sorted out by the following GO function.

Gene symbol	Gene name	GO
CTGF	connective tissue growth factor	1-6
TNFRSF12A	tumor necrosis factor receptor superfamily, member 12a	1-5
PDGFRB	platelet derived growth factor receptor, beta polypeptide	1,5
RHOB	ras homolog gene family, member B	1-3
THBS1	Thrombospondin 1	1,3,6
RTN4	reticulon 4	1-3
ANXA2	annexin A2	1,6
ARHGAP24	Rho GTPase activating protein 24	1
APP	amyloid beta (A4) precursor protein	2,3,5,6
CD44	CD44 antigen	2,3,5,6
COL1A1	procollagen, type 1, alpha 1	2,3,5,6
EDNRB	endothelin receptor type B	2-6
GJA1	gap junction membrane channel protein alpha 1	2-5
LAMC1	laminin, gamma 1	3,5,6
PPP2CA	protein phosphatase 2 (formerly 2A), catalytic subunit, alpha isoform	2,3,5
SPP1	secreted phosphoprotein 1	2,3,5,6
LOC500040	similar to Testis derived transcript	3-5
FATH	fat tumor suppressor homolog	3,5

GPNMB	glycoprotein (transmembrane) nmb	3,5
CCNA2	cyclin A2	3,5
CD36	cd36 antigen	3
CD24	CD24 antigen	3
CXADR	coxsackie virus and adenovirus receptor	3
DDR1	discoidin domain receptor family, member 1	3
DCN	decorin	3,6
DPYSL2	dihydropyrimidinase-like 2	3
ENPP1	ectonucleotide pyrophosphatase/phosphodiesterase 1	3
EPDR2	ependymin related protein 2 (zebrafish)	3
LGALS2	lectin, galactoside-binding, soluble 2	3
SPINT1	serine peptidase inhibitor, Kunitz type 1	3
TACSTD1	tumor-associated calcium signal transducer 1	3
TM4SF3	transmembrane 4 superfamily member 3	3
TMSB10	thymosin, beta 10	3
ITGA6	integrin, alpha 6	2,3
SDCBP	syndecan binding protein	3,4
TNS	tensin	3,4
CFL1	cofilin 1, non-muscle	2,4
MGP	matrix Gla protein	3,6
NID1	Nidogen 1	3,6
VIL2	villin 2	3

TSPAN3	tetraspanin 3	3,5
IGFBP3	insulin-like growth factor binding protein 3	2,5
EGR1	early growth response 1	2,5
EGLN3	EGL nine homolog 3 (C. elegans)	2,5
GAS6	growth arrest specific 6	2,5
MYC	myelocytomatosis viral oncogene homolog (avian)	2,5
MTAP2	microtubule-associated protein 2	2,5
NME1	expressed in non-metastatic cells 1	2,5
ANXA5	annexin A5	2
BZRP	benzodiazepine receptor, peripheral	2
CD74	CD74 antigen	2,5
BEX1	brain expressed X-linked 1	2,5
TRIB3	tribbles homolog 3 (Drosophila)	2
CRYAB	crystallin, alpha B	2
LITAF	LPS-induced TN factor	2
DDIT4	DNA-damage-inducible transcript 4	2
FCGR3	Fc receptor, IgG, low affinity III	2
G6PDX	glucose-6-phosphate dehydrogenase X-linked	2
GCLC	glutamate-cysteine ligase, catalytic subunit	2
GGT1	gamma-glutamyltransferase 1	2
HSPCB	heat shock 90kDa protein 1, beta	2
ABCB1A	ATP-binding cassette, sub-family B (MDR/TAP), member 1A	2

AKR1B4	aldo-keto reductase family 1, member B4	2
AQP1	aquaporin 1	2
BTG2	B-cell translocation gene 2, anti-proliferative	2
NQO1	NAD(P)H dehydrogenase, quinone 1	2
NEK6	NIMA (never in mitosis gene a)-related expressed kinase 6	2
NGFRAP1	nerve growth factor receptor (TNFRSF16) associated protein 1	2
NOL3	nucleolar protein 3 (apoptosis repressor with CARD domain)	2
YWHAQ	tyrosine 3-monooxygenase	2
PRNP	prion protein	2
SLC25A4	solute carrier family 25	2
TFRC	transferrin receptor	2
VIM	vimentin	2
TUBA1	tubulin, alpha 1	2,5
LGALS3	lectin, galactose binding, soluble 3	2,6
IGFBP7	insulin-like growth factor binding protein 7	5
CCND1	cyclin D1	5
S100A6	S100 calcium binding protein A6	5
SPARC	secreted acidic cysteine rich glycoprotein	5,6
DMBT1	deleted in malignant brain tumors 1	5,6
HSPCA	heat shock protein 1, alpha	5
EMP1	epithelial membrane protein 1	5
DAB2	disabled homolog 2 (Drosophila)	5

ANXA1	annexin A1	5
ANXA7	annexin A7	5
ATPIF1	ATPase inhibitory factor 1	5
S100A11	S100 calcium binding protein A11	5
CD276	CD276 antigen	5
TAX1BP3	Tax1 (human T-cell leukemia virus type I) binding protein 3	5
TXNRD1	thioredoxin reductase 1	5
NPDC1	neural proliferation, differentiation and control, 1	5
PKIB	protein kinase inhibitor beta, cAMP dependent, catalytic	5
COL1A2	procollagen, type I, alpha 2	6
COL3A1	procollagen, type III, alpha 1	6
COL4A1	procollagen, type IV, alpha 1	6
COL5A2	procollagen, type V, alpha 2	6
ACP5	acid phosphatase 5, tartrate resistant	6
LTBP1	latent transforming growth factor beta binding protein 1	6
DEFB1	defensin beta 1	6

NOTE: The numbers from 1-6 indicate GO terms: angiogenesis, apoptosis, cell adhesion, cell migration, cell proliferation and extracellular matrix, respectively.
